# Loss of CNF_Y_ toxin-induced inflammation drives *Yersinia pseudotuberculosis* into persistency

**DOI:** 10.1371/journal.ppat.1006858

**Published:** 2018-02-01

**Authors:** Wiebke Heine, Michael Beckstette, Ann Kathrin Heroven, Sophie Thiemann, Ulrike Heise, Aaron Mischa Nuss, Fabio Pisano, Till Strowig, Petra Dersch

**Affiliations:** 1 Department of Molecular Infection Biology, Helmholtz Centre for Infection Research, Braunschweig, Germany; 2 Group Microbial Immune Regulation, Helmholtz Centre for Infection Research, Braunschweig, Germany; 3 Group Mouse Pathology, Helmholtz Centre for Infection Research, Braunschweig, Germany; Stanford University School of Medicine, UNITED STATES

## Abstract

Gastrointestinal infections caused by enteric yersiniae can become persistent and complicated by relapsing enteritis and severe autoimmune disorders. To establish a persistent infection, the bacteria have to cope with hostile surroundings when they transmigrate through the intestinal epithelium and colonize underlying gut-associated lymphatic tissues. How the bacteria gain a foothold in the face of host immune responses is poorly understood. Here, we show that the CNF_Y_ toxin, which enhances translocation of the antiphagocytic Yop effectors, induces inflammatory responses. This results in extensive tissue destruction, alteration of the intestinal microbiota and bacterial clearance. Suppression of CNF_Y_ function, however, increases interferon-γ-mediated responses, comprising non-inflammatory antimicrobial activities and tolerogenesis. This process is accompanied by a preterm reprogramming of the pathogen's transcriptional response towards persistence, which gives the bacteria a fitness edge against host responses and facilitates establishment of a commensal-type life style.

## Author summary

A complex cascade of events is triggered by the mammalian host upon a bacterial infection to prevent pathogen-induced damage. Many pathogens are successfully eliminated by the host immune system. However, some bacterial pathogens, including enteric yersiniae, evolved strategies to efficiently evade immune surveillance. This enables the pathogen to colonize specific sites in the host in which they can persist for years. These persistent infections are often asymptomatic for long time periods, but can undergo reactivation and cause clinical symptoms. In order to understand how enteric bacteria can persist in gut-associated host niches, we investigated changes of bacterial and host responses from an acute to a persistent infection. We found that persistency of *Y*. *pseudotuberculosis* in the cecum is characterized by suppression of pathogen-induced inflammation and tissue destruction, and an induction of interferon-γ promoted tolerogenic responses. We further show that the removal of a single bacterial virulence factor, the CNF_Y_ toxin, can trigger this process and drives the bacteria into persistency.

## Introduction

Infections by bacterial pathogens generally result in induction of host immune responses and the development of acute disease. Many of these intruders are successfully cleared by the host immune system. However, some evolved strategies to efficiently evade immune responses, enabling the pathogen to persist for long periods in preferential host niches [1]. Some persistent infections result in clinically apparent chronic symptoms, e.g. chronic inflammation and autoimmunity [2]. In other cases, persistent infections are asymptomatic for decades before they undergo reactivation with severe clinical symptoms [3].

Also gastrointestinal infections caused by enteric yersiniae, *Shigella* and salmonellae, can become persistent and complicated by the development of severe autoimmune disorders [4]. The predominant forms of *Yersinia pseudotuberculosis* and *Y*. *enterocolitica* infections in humans are usually self-limiting gastrointestinal disorders like enteritis, diarrhea and mesenteric lymphadenitis, termed Yersiniosis, but they occasionally lead to autoimmune disorders like erythema nodosum [5]. Evidence exists that *yersiniae* can persist silently in the intestinal mucosa and the lymphoid tissue of the submucosa of humans for several years, causing chronic ileitis, relapsing enteritis and the development of reactive arthritis [6]. Why and how enteropathogenic *yersiniae* can persist in some patients is unknown.

Mouse models, displaying acute disease symptoms similar to humans, revealed that the bacteria colonize the distal ileum and proximal colon, and enter the Peyer’s patches from which they spread directly or via the mesenteric lymph nodes to liver and spleen [7, 8]. Recently, a murine infection model for persistent *Y*. *pseudotuberculosis* infection was established. Sublethal infection resulted in prolonged asymptomatic colonization of the cecum and shedding of *Yersinia* with the feces in a fraction of mice (10–25%) [9]. This suggested that the cecum is a beneficial reservoir for dissemination to extra-intestinal sites. Elevated cytokine levels in the serum further indicate circulating antigens during the persistent state, which could promote/support the development of reactive arthritis.

Previous studies to identify the mechanisms enabling *Y*. *pseudotuberculosis* persistence in the cecum revealed that the bacteria undergo a profound transcriptional reprogramming from the acute to the persistent stages of infection [10]. Several functions, i.e. for anaerobic growth, motility, and protection against host stress are induced, whereas 466 genes were found to be > 2-fold downregulated during the persistence stage. Among them are important acute stage virulence genes, including the adhesins Ail and YadA, the cytotoxic necrotizing factor CNF_Y_, the virulence plasmid-encoded type III secretion system (T3SS) and the associated Yop effectors, which are highly upregulated during the initial phase of the infection [10]. The Yop effectors prevent phagocytosis by leukocytes, which is important for colonization and systemic dissemination [11]. The CNF_Y_ toxin constitutively activates small Rho GTPases by deamidation and improves Yop translocation into host cells, thereby enhancing inflammation and tissue damage [12, 13].

In the present study, we further demonstrate that suppression of CNF_Y_ function shifts the balance of bacteria-triggered inflammation and clearance mechanisms towards induction of immune suppression, promoting the establishment of asymptomatic, persistent infection. An evaluation of the *Y*. *pseudotuberculosis* expression program of a *cnfY* mutant further revealed early reprogramming from virulent to persistent mode, which triggers a host response that allows a commensal-type life-style.

## Results

### Absence of CNF_Y_ enhances *Yersinia* persistence

In our previous work we found that absence of a functional CNF_Y_ toxin decreases the pathogenicity of the *Y*. *pseudotuberculosis* wildtype strain YPIII [12]. Mice that were infected at high doses (2x10^9^ CFU) with the isogenic Δ*cnfY* mutant survived the infection compared to wildtype-infected mice, yet carried high numbers of the bacteria in the gut compartments and associated lymphatic tissue. Another study demonstrated that expression of *cnfY* is downregulated in *Y*. *pseudotuberculosis* persistently residing in the cecum of mice [10]. Based on these results we speculated that absence of the CNF_Y_ toxin supports the establishment of persistent, asymptomatic infection.

**Fig 1:**
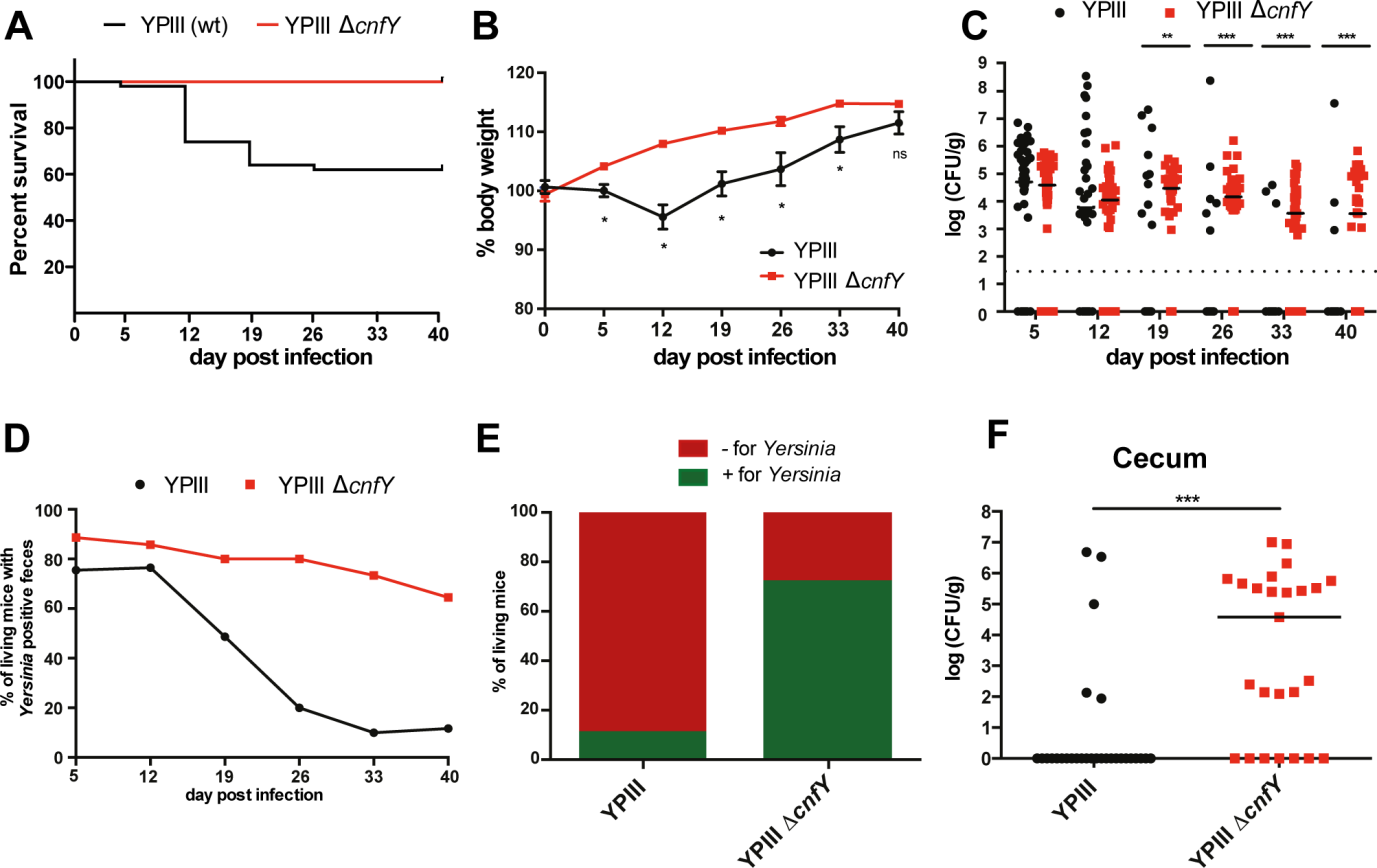
Deletion of *cnfY* allows establishment of a persistent infection. BALB/c mice were infected with 10^6^ CFU of YPIII (n = 50) or YP147(Δ*cnfY*) (n = 25). (**A**) Survival of mice infected with YPIII or YP147(Δ*cnfY*). (**B**) The body weight of surviving mice was monitored over 6 weeks. The mean ±SEM from 3 independent experiments is shown; YPIII, or YP147(Δ*cnfY*). The statistical significance was determined by multiple t-tests, Holm-Šídák correction. P-value: * <0.05. (**C**) The *Yersinia* burden in the feces of YPIII- or YPIII Δ*cnfY*-infected mice was determined. The median represents two independent experiments. The statistical significance was determined by the Mann-Whitney U test. P-values: ** <0.01, *** <0.001. (**D**) Percentage of mice positively tested for *Yersinia* in the feces of YPIII or YP147(Δ*cnfY*). Percentage (**E**) and the bacterial burden (**F**) of mice positively tested for YPIII (n = 31) or YP147(Δ*cnfY*) (n = 25) in the cecum at day 42. The median of three independent experiments is shown. Mann-Whitney U test was used for statistical analysis. P-value: *** <0.001.

To investigate the function of the CNF_Y_ toxin in the progression of a persistent bacterial infection, BALB/c mice were orally infected using a low infection dose (10^6^ CFU) of *Y*. *pseudotuberculosis* wildtype or the Δ*cnfY* mutant and the infection was followed for 6 weeks. Approximately 40% of the wildtype-infected mice succumbed to infection between day 5 to day 26 (**Fig 1A**). Weight loss analysis demonstrated that the remaining 60% of the mice that survived infection displayed an average weight loss of 4% in the first two weeks of infection (Fig **1B**). In contrast, mice infected with the Δ*cnfY* mutant showed no weight loss during the course of the infection (Fig **1B**). To obtain information about the persistence of the bacteria in the intestine, we assessed the load of *Yersinia* in the feces. The overall number of wildtype bacteria in the stool samples decreased rapidly after 2 weeks post infection and indicated clearance of the bacterium in approximately 90% of the infected mice at 40 dpi (Fig **1C and 1D**). This result was supported by the analysis of the percentage of mice, which still contained bacteria in their ceca 42 dpi (Fig **1E**). In contrast, the Δ*cnfY* mutant was detectable in the feces and ceca of almost 70% of mice at the end of the experiment and the number of bacteria remained fairly stable in the individual mice (Fig **1C–1E**). Quantification of the bacteria revealed that 50% of all Δ*cnfY* mutant-infected mice, which were still colonized, contained more than 10^4^ CFU/g tissue (Fig **1F**). In contrast, less than 10% of the mice which were infected with the wildtype contained >10^4^ CFU/g tissue (Fig **1F**). Altogether, these data show that absence of the CNF_Y_ toxin impairs bacterial clearance and promotes establishment of persistent infection by *Y*. *pseudotuberculosis* in cecal tissue.

### *Y*. *pseudotuberculosis* residual in ceca escapes the immune response

**Fig 2:**
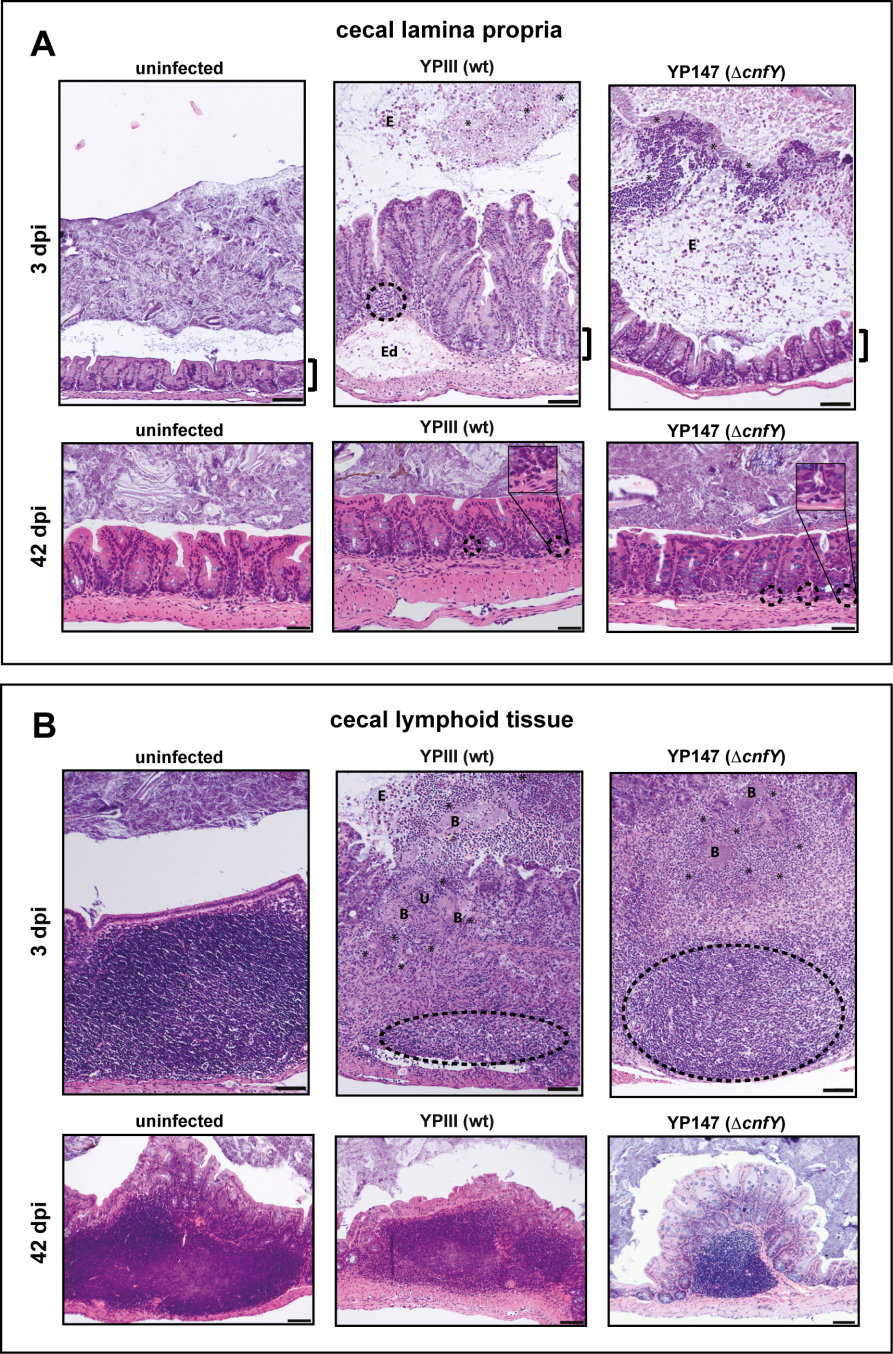
Tissue alterations during acute and persistent infection of the cecum. H&E stained sections of the cecal lamina propria (**A**) and the cecal lymphoid tissue (**B**) of BALB/c mice at 3 or 42 dpi with about 10^5−^10^6^ CFUs of YPIII or YP147(Δ*cnfY*)*/*g tissue, or uninfected mice. Cecal lamina propria (3 dpi); YPIII: focal invasion of lymphocytes into the lamina propria (dashed halo) and edema formation (Ed). YP147(Δ*cnfY*): diffuse distributed granulocytes. E: epithelial cells. Cecal lamina propria (42 dpi); YPIII and YP147(Δ*cnfY*): isolated granulocytes at the basal lamina propria (dashed halo). Cecal lymphoid tissue (3 dpi); YPIII: massive necrosis, destroyed follicles (dashed halo), ulcus formation (U) and bacterial microcolonies (B) surrounded by invaded granulocytes (black asterisks). E: epithelial cells. YP147(Δ*cnfY*): necrotic parts and reduced lymphocytes in follicle (dashed halo). Infiltrating granulocytes surround bacterial microcolonies (black asterisks). Pictures show representatives of multiple fields of sections from groups of 3–5 mice. The brackets illustrate the length of the microvilli of the uninfected mice during the acute infection phase. Bar: (**A**) 50 μm, lower panel, 100 μm upper panel, (**B**) 100 μm.

Secretion of CNF_Y_ by *Y*. *pseudotuberculosis* was shown to trigger inflammation in the ileum during acute infection [12]. Since a persistent infection is more efficiently established in the absence of CNF_Y_, we addressed whether the development of persistence is accompanied by distinct inflammatory reactions in the cecum. Infection with the wildtype caused a very severe inflammation in the cecum during early acute phase (3 dpi) (**S1** Fig). The inflammation was diffuse and affected the entire lamina propria and the cecal lymphoid follicles (Fig **2**). Histopathological examinations of wildtype-infected tissue revealed an elongation of the villi length due to epithelial cell hyperplasia, a high degree of edema formation, and a massive infiltration of lymphocytes, whereas inflammation in Δ*cnfY* mutant-infected mice was less severe and was mainly characterized by diffuse infiltrated polymorphonuclear leukocytes (PMNs) in the lamina propria (Fig **2A**). Numerous bacterial foci surrounded by infiltrated PMNs were detected in the cecal lymphoid follicles of wildtype and Δ*cnfY* mutant-infected mice 3 dpi (Fig **2B**). Although the number of the bacterial foci was comparable, inflammation was much more severe in cecal lymphoid follicles of the wildtype-infected mice. A stronger superficial destruction of the epithelial lining (ulcus formation), and a much more drastic tissue remodeling with necrosis was observed (Fig **2B**).

In contrast to the acute infection, no obvious alterations of the lamina propria and the cecal lymphoid tissue were detected between the wildtype- and Δ*cnfY* mutant-infected mice 42 dpi (Fig **2**). Only very mild signs of infection (i.e. diffuse, local infiltration of isolated granulocytes) were detectable in the basal part of the lamina propria in mice infected with the wildtype or the Δ*cnfY* mutant (Fig **2A**). This indicates that *cnfY*-deficient *Y*. *pseudotuberculosis* has the capacity to persist in the cecal tissues avoiding recognition and destruction by the immune system.

### *Yersinia* colonization pattern is altered in persistent stage

*Y*. *pseudotuberculosis* is primarily an extracellular pathogen, that grows in large bacterial microcolonies in infected tissue early after infection [14]. Given the high bacterial burdens in the cecal tissue of the *cnfY* mutant in the persistent stage (Fig **1F**), it was surprising that we were unable to identify bacterial microcolonies in the tissue, as was the case during acute phase (Fig **2B**). This indicated that the localization and/or distribution of the bacteria differ between the acute and the persistent phase. To investigate this issue, we used constitutively *mRuby2*-expressing *Yersinia* strains (YPIII *mRuby2*, YP147(Δ*cnfY*) *mRuby2*), which show no changes in growth, virulence and development of persistence (**S2** Fig), to follow their localization.

**Fig 3:**
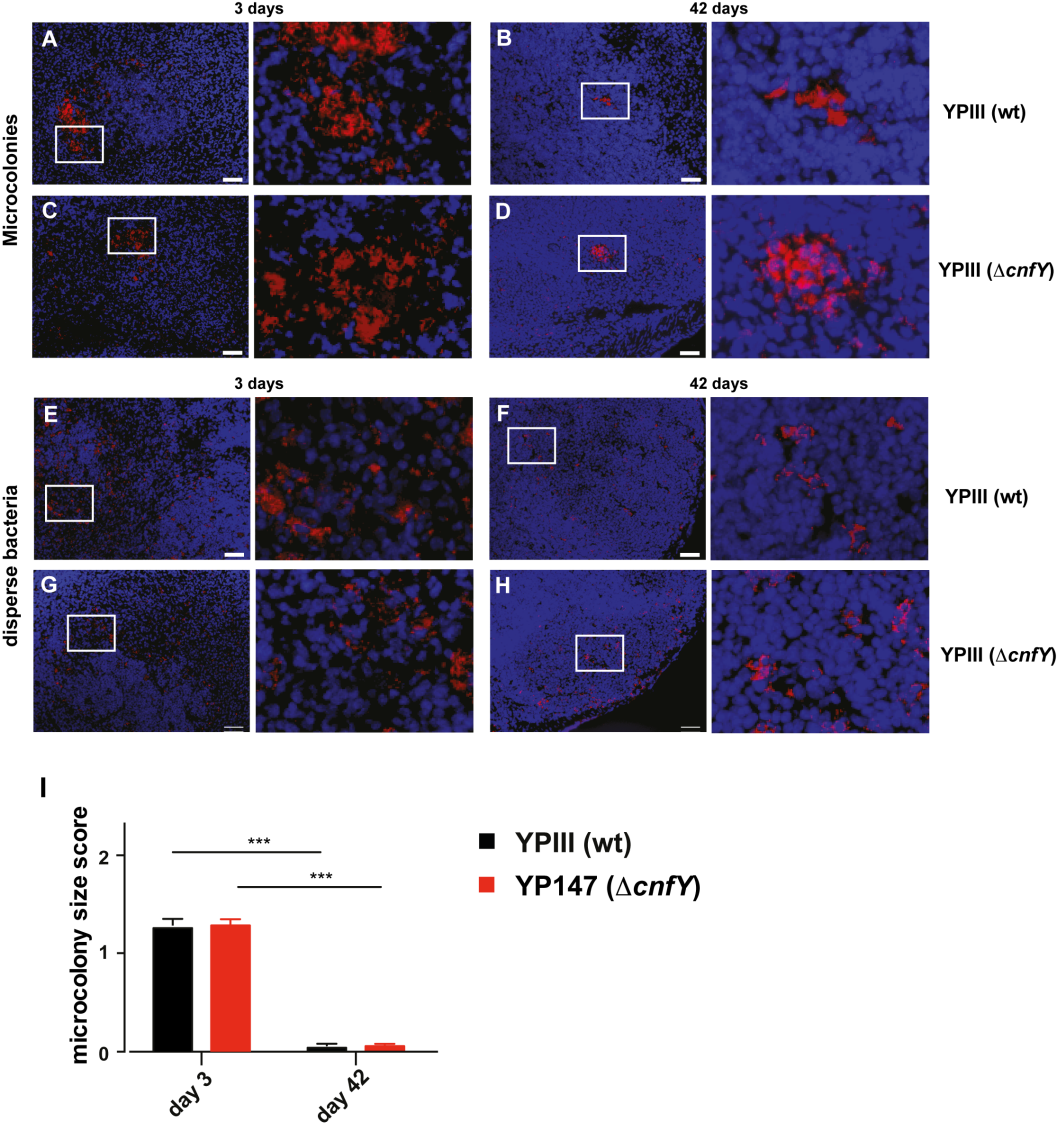
Colonization patterns of *Y*. *pseudotuberculosis* during acute and persistent infection. Microscopic detection of mRuby2-expressing *Yersinia* in cecal lymphoid tissue of infected BALB/c mice at 3 and 42 dpi with approximately 10^5^−10^6^ bacteria/g tissue. Blue: DAPI-stained host cell nuclei; red: mRuby2-expressing bacteria. Bar: 50 μm. Microcolonies of YPIII (*mRuby2*) 3 dpi (**A**) or 42 dpi (**B**), YP147(Δ*cnfY*) (*mRuby2*) 3 dpi (**C**), or 42 dpi (**D**). Disperse bacteria of YPIII (*mRuby2*) 3 dpi (**E**) or 42 dpi (**F**), YP147(Δ*cnfY*) (*mRuby2*) 3 dpi (**G**) or 42 dpi (**H**). Representatives of multiple sections from groups of 3 mice are shown. (**I**) Scoring of the *Yersinia* colonization pattern of multiple microscopic sections of YPIII (*mRuby2*)- or YP147(Δ*cnfY*) (*mRuby2*)-infected mice (3 mice/group) at 3 or 42 dpi (Supplemental Information). Number of scored microscopy fields for microcolonies 3 dpi: YPIII n = 141, YP147(Δ*cnfY*) n = 237; 42 dpi: YPIII n = 166, YP147(Δ*cnfY*) n = 167. Data show the mean of scores ±SEM. Statistical analysis was performed using multiple t-tests, Holm-Šídák correction; P-values: * <0.05, ** <0.01, *** <0.001.

The colonization pattern of YPIII *mRuby2* and YP147(Δ*cnfY*) *mRuby2* in the cecal lymphoid tissue were compared 3 and 42 dpi. During the early infection many large microcolonies within prominent necrotic areas or severe lesions (Fig **3A and 3C**) as well as multiple dispersedly distributed single bacteria and few-cell aggregates (Fig **3E and 3G**) were observed in the cecal lymphoid tissue of both infection groups. No major differences in the overall sizes of the large microcolonies (Fig **3I**) or necrotic lesions (Fig **3A and 3C**) were detectable.

A strikingly different pattern was observed during persistent infection, although the overall number of bacteria in analyzed cecal tissue was comparable (10^5^−10^6^ CFU/g). Only occasionally, we found single very densely packed microcolonies in both groups (Fig **3B, 3D and 3I**). In contrast to the acute phase, bacteria were predominantly visualized as single cells or in aggregates containing few cells in undamaged tissue, residing in the intercellular space surrounding lymphocytes (Fig **3F and 3H**). Similar colonization patterns of the wildtype and the Δ*cnfY* mutant indicated that the distinct colonization behavior in the persistent stage is independent of CNF_Y_.

### Secretion of CNF_Y_ alters the composition of the microbiota

**Fig 4:**
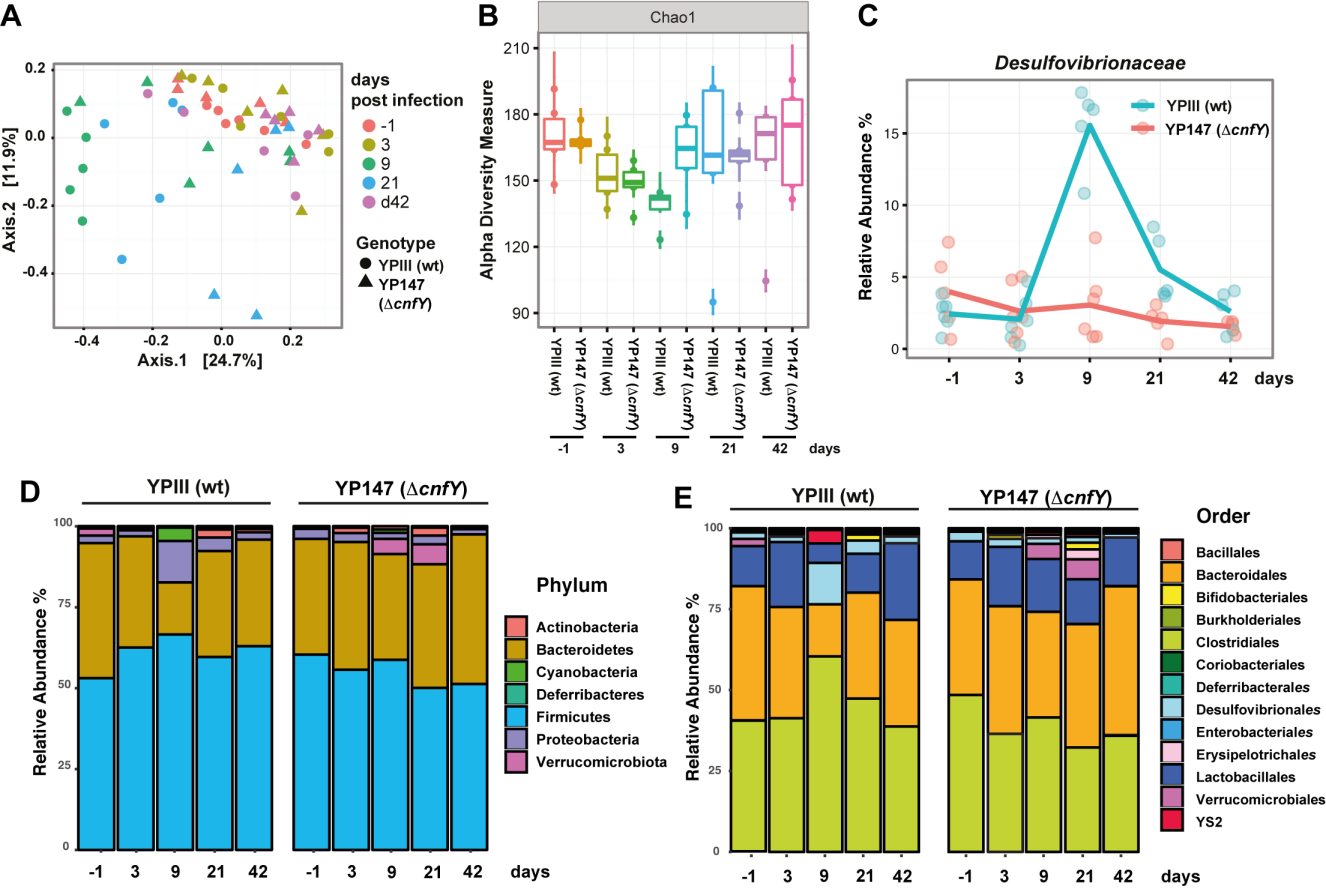
Gut microbiota in wildtype- and Δ*cnfY* mutant-infected mice. At indicated time points prior (-1) and post infection, feces was sampled from individual mice and tested for *Y*. *pseudotuberculosis*. The microbiota composition of persistently *Yersinia*-infected mice was analyzed by 16S rRNA gene sequencing. (**A**) Principal coordinates analysis (PCoA) was used to visualize β diversity globally and the bar plot displays the contribution of variables to the observed variance over all time points. (**B**) Analysis of α diversity using Chao1 index. (**C**) Relative abundance of the families *Desulfovibrionaceae* at indicated time points. Relative abundance of the bacteria grouped taxonomically by phyla (**D**) or microbial orders (**E**) from 5–6 mice.

Induced inflammation in the intestinal tract by enteric pathogens was shown to alter the composition of the residual microbiota and influence the outcome and persistence of the infection [15]. As several studies describe that also *Yersinia*-triggered inflammation of the intestine leads to global alterations of the commensal microflora [16, 17], we tested whether the Δ*cnfY* mutant induced a change of the commensal population. To do so, the composition of the intestinal microbial community in the feces of wildtype and Δ*cnfY* mutant-infected mice was determined by 16S rRNA gene sequencing from stool samples during the course of the infection. Communities in individual mice were compared by principal components analysis using Bray-Curtis dissimilarity distances. We used permutational multivariate analysis of variance (ADONIS) [18], considering the strain and day of infection to evaluate their relative contribution to variability within the microbiota. As shown in Fig **4A, 4D and 4E** we observed a significant shift in the microbiota during the course of infection (R2 = 0.18), which was most prominent in wildtype-infected mice 9 dpi. Notably, the relative abundance of Phyla recovered largely to pre-infection levels in the persistent state (42 dpi) (Fig **4A–4E**). The species richness within the community (α diversity described by the Chao1 index) did not change significantly during the infection (Fig **4B**), suggesting that changes in the relative abundance of distinct bacterial groups are responsible for the changes in the composition of the microbiota. Loss of CNF_Y_ had a small, but measurable effect on microbiota composition in the global analysis (R^2^ = 0.04 considering all time points). A more detailed analysis of the communities at the different time points after infection (**S3A–S3F** Fig) further showed that presence of CNF_Y_ had a larger influence on microbiota composition during establishment of the persistent infection (day 9: R^2^ = 0.24, day 21: R^2^ = 0.22 and day 42: R^2^ = 0.24). As severe inflammation of the cecal tissue was already observed at 3 dpi (Fig **2**), when no changes of the microbiota were detectable, it is assumed that changes of the microbiome are a consequence of the inflammation.

In the wildtype-infected mice, we observed an increase in the relative abundance of the Proteobacteria (Desulfovibrionales) 9 dpi, whereas other orders such as Bacteroidales and Lactobacillales, connected to anti-bacterial and anti-inflammatory effects [19], seem reduced compared to uninfected mice. In particular sulfate-reducing bacteria of the family *Desulfovibrionaceae* were significantly induced (Fig **4C**), which were previously shown to be associated with inflammation in mice [20]. In contrast, only a very mild change, e.g. in the relative abundance of the Bacteroidetes and Verrucomicrobiota, was observed in the Δ*cnfY* mutant-infected mice (Fig **4D and 4E**). These data illustrate that the degree of induced alterations of the commensal microbiota is not linked to the luminal colonization of *Yersinia*. It is rather a consequence and correlates with the severity and/or nature of *Yersinia*-triggered inflammation, which is affected by the presence of CNF_Y_.

### Absence of the CNF_Y_ toxin triggers a distinct host response

The establishment of a persistent infection with the Δ*cnfY* mutant might be the result of a dampened inflammatory response during the acute phase. In order to explore host immune reactions underlying the establishment of *Yersinia* persistence and to assess how CNF_Y_ impacts this process, we employed a strand-specific RNA-seq approach to determine the host transcriptome of mice infected with the wildtype and the Δ*cnfY* mutant during acute and persistent infection (**S4A** Fig).

For this purpose, high quality RNA pools isolated from ceca of uninfected or infected mice (**S4B** Fig) colonized with equal numbers of wildtype and Δ*cnfY* bacteria (**S4C** Fig) were depleted for mouse rRNA to increase coverage of informative mouse transcripts. A set of RNA standards was added to the individual RNA pools to judge the accuracy of determined fold changes between replicates and the mixture was used for cDNA library preparation (for details see Supplemental Information). Strand-specific Illumina-based deep sequencing of the cDNA generated approximately 18–40 million cDNA reads per sample of which around 10–24 million mapped to the mm10 genome (**S1** Table). **S5A** Fig shows that read density and RNA input correlated strictly over the entire detection range of 18 log_2_ units concentration, and the linear fit shown in **S5B** Fig documents highly accurate fold-change estimates. Moreover, all samples correlated closely with their respective replicates (**S6** Fig). Sufficient coverage and high quality of our data enabled us to reliably quantify transcript abundance and compare the host global expression profiles under the different conditions.

**Fig 5:**
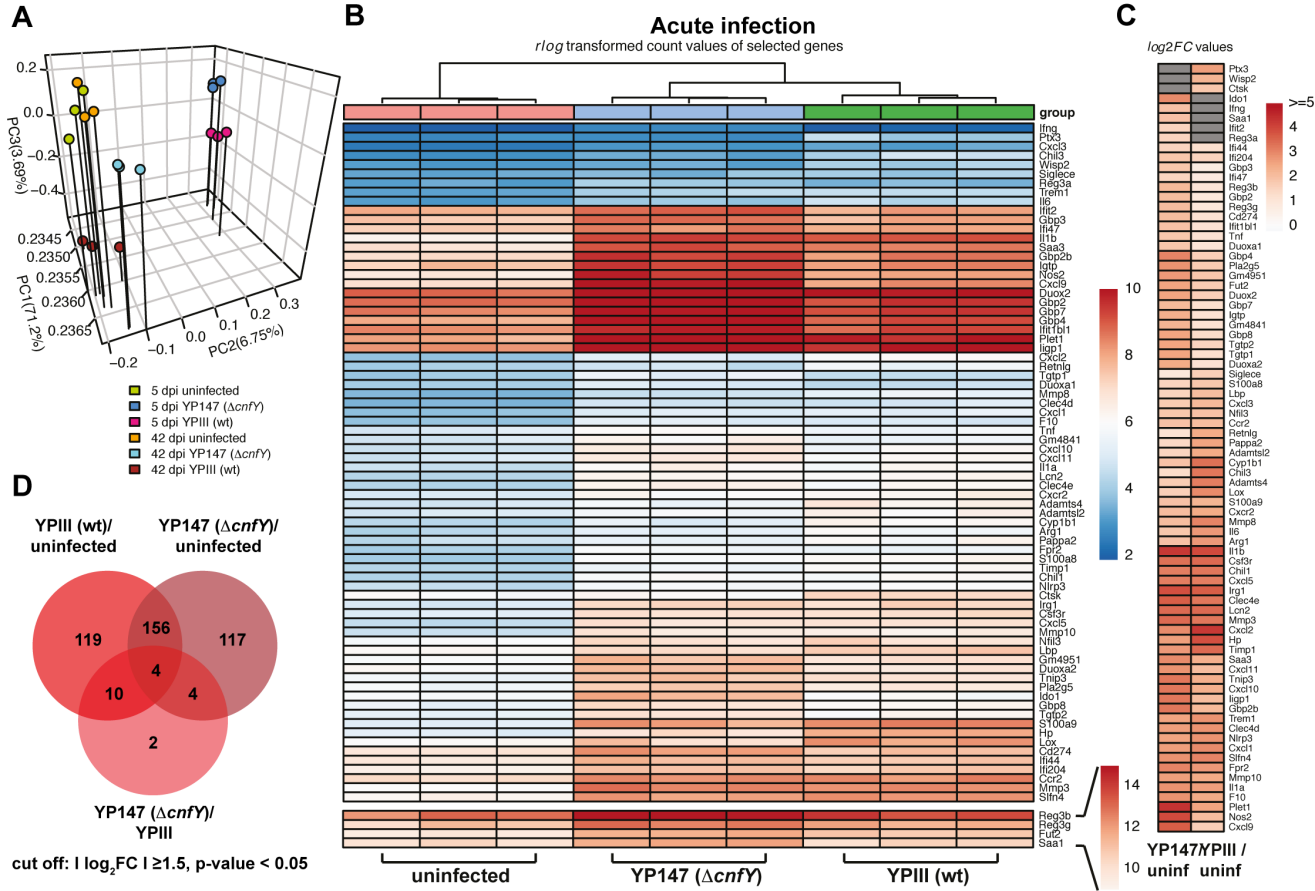
Host transcriptome of *Y*. *pseudotuberculosis-*infected ceca. (**A**) Principal component analysis (PCA) of mean centered and scaled *rlog* transformed read count values of tissue RNA-seq data of uninfected and *Yersinia*-infected mice. (**B**) Heat map of the top enriched host transcripts based on *DESeq*2 analyses. Color-coding is based on *rlog* transformed read count values. (**C**) Heat map illustrates log2 fold changes of host transcripts detected in YPIII- or YP147(Δ*cnfY*)-infected mice compared to uninfected mice (adjusted P value ≤ 0.05). Grey boxes: not significant. (**D**) Venn-diagram of differentially expressed genes from uninfected versus YPIII or YP147(Δ*cnfY*) 5 dpi.

The global gene expression profiles of uninfected and infected ceca were distinct and independent replicates clustered together (Fig **5A**). Hierarchical clustering of wildtype- and Δ*cnfY* mutant-infected ceca profiles further emphasized that the host exerts a different response towards both strains during acute infection. The expression profiles of persistently infected ceca cluster close to the profiles of uninfected ceca, illustrating that the difference between the transcriptomes is less substantial (Fig **5A**).

### Tissue RNA-seq highlights protective immune-mechanisms

To gain insight into the *Yersinia*-induced host reactions, including all immune response-specific stimuli that may impact development and maintenance of the persistent state, we employed the differential expression analysis package DESeq2 [21]. Of the >17,000 profiled host transcripts in wildtype- and Δ*cnfY* mutant-infected ceca isolated at 5 dpi, 178 and 204 transcripts were more abundant (log_2_FC ≥ 1.5) and 111 and 77 transcripts were less abundant (log_2_FC ≤ -1.5) than in uninfected mice (**S7A** Fig, **S1** and **S2** Datasets).

To identify important infection-linked pathways and functions, we performed Gene Ontology and KEGG pathway enrichment analysis with the list of identified differentially expressed genes (**S2** Table). Among the top enriched cellular pathways and processes were many involved in immune responses, which have recently been described in the Peyer's patches during a *Y*. *pseudotuberculosis* IP32953 infection [22]. Multiple host immune responses were induced by both, the wildtype and the Δ*cnfY* mutant strain, during the acute phase, whereas others were only induced in wildtype- or Δ*cnfY* mutant-infected tissue (Fig **5B and 5C**, **S1** and **S2** Datasets). Of the 160 commonly regulated candidates were genes of major proinflammatory cyto- and chemokines (e.g. IL-1α/β, IL-6, Cxcl1, Cxcl2, Cxcl3, Cxcl5, Cxcl9, Cxcl10, Ccl2, Ccl3), their receptors (e.g. Csf3r, Trem1, Cxcr2, Ccr2, Ccr5), and inflammasome, superoxide generating, chemotactic, and signaling factors (e.g. Irg1, Nlrp3, Fpr2, Nfil3) (Fig **5B**, **S1** and **S2** Datasets). They are implicated in the recruitment, differentiation and activation of inflammatory cells (in particular neutrophils) to infected and damaged tissue [23–25]. In addition, transcripts of components of the acute phase response and fibrinolysis important for the clearing of pathogens and healing of damaged tissue (e.g. matrix metalloproteinases and inhibitors: Mmp3, Mmp8, Mmp10, Timp1, Saa3, Clec4e, Clec4d, Chil1, F10, Plet1) [26, 27] reactive compound protection (cytochrome P450/Cyp1b1, Arg1) [28, 29], as well as metal ion scavenging proteins (e.g. haptoglobin, lipocalin, calprotectin S100A8/9) were strongly enriched during the infection with both *Y*. *pseudotuberculosis* strains.

Importantly, many of the proinflammatory responses and defense functions were stronger induced in the wildtype-infected tissues or not expressed in the Δ*cnfY* mutant-infected ceca (e.g. IL-6, Cxcl2, Mmp8, Chil3, cytochrome P450/Cyp1b1, Arg1, haptoglobin, Ptx3). In addition, transcripts for extracellular matrix modeling and tissue architecture modifying enzymes (such as Adamts4, Adamtsl2, Lox, Wisp2/CCN5, Pappa2, Retnlg), which are involved in tissue damage repair and regeneration, cell proliferation and barrier maintenance [30–34] are also more abundant in wildtype-infected tissue (Fig **5B and 5C**, **S1**–**S3** Datasets). In agreement with previous data this demonstrates that certain inflammatory responses and induced tissue damage are considerably stronger in the presence of CNF_Y_.

We further observed that about 43% (121 transcripts) of all differentially regulated genes in Δ*cnfY* mutant-infected mice are not in- or reduced in the wildtype-infected animals (Fig **5D**). This includes many transcripts implicated in the control of adaptive immune responses. Among them are interferon-γ (ifng) and multiple interferon-induced genes (Ifit2, Ifi44, Ifi47, Ifi204, Ifit1bl1, Igtp) as well as the tumor necrosis factor (tnf), which stimulates host defense (Fig **5B and 5C**). One of the most upregulated factors was the indoleamine 2,3-dioxigenase (Ido1). Ido1 is an immune checkpoint enzyme that enforces depletion of tryptophan by conversion to kynurenine derivatives. This protects the host from over-reactive effector T cells via induction of immunosuppression and the onset of tolerogenesis [35]. Both functions seem to dampen the immune response mediating immune-escape of the bacteria. Moreover, many other enriched transcripts were involved in T cell chemoattraction, activation, survival, differentiation, and modulation (Ly6a, Saa1, Saa2, CD274, IFN-γ inducible ligands: Cxcl9, Cxcl10, Cxcl11) [36, 37]. In addition, multiple genes encoding GTP binding proteins of the cell autonomous immunity (Iigp1, Gbp2/2b/3/4/7/8, Igtp, Ifgga2(GM4951), Ifgga3(Gm4841) [38] as well as factors that possess bactericidal/bacteriostatic activities (i.e. Nos2, Duox2, Duoxa2, Pla2g5, Reg3b/g) [39, 40] were strongly induced in Δ*cnfY*-infected ceca (Fig **5B and 5C**, **S1**–**S3** Datasets). In parallel, many factors were upregulated that promote cell differentiation and establishment of epithelial layers (Plet1) and limit inflammation, e.g. Tnip3 **(**Abin3) inhibiting NF_K_B-induced TLR4 and IL-1 responses [41, 42] (Fig **5B and 5C**, **S1**–**S3** Datasets).

In summary, the results imply that the wildtype triggers an exacerbated inflammatory response and tissue damage to clear the infection, whereas the Δ*cnfY* mutant induces responses leading to a more cell autonomous and tolerogenic immunity that render the host more competent to permit a persistent infection.

### Minor changes of the host transcriptome during persistent infection

**Fig 6:**
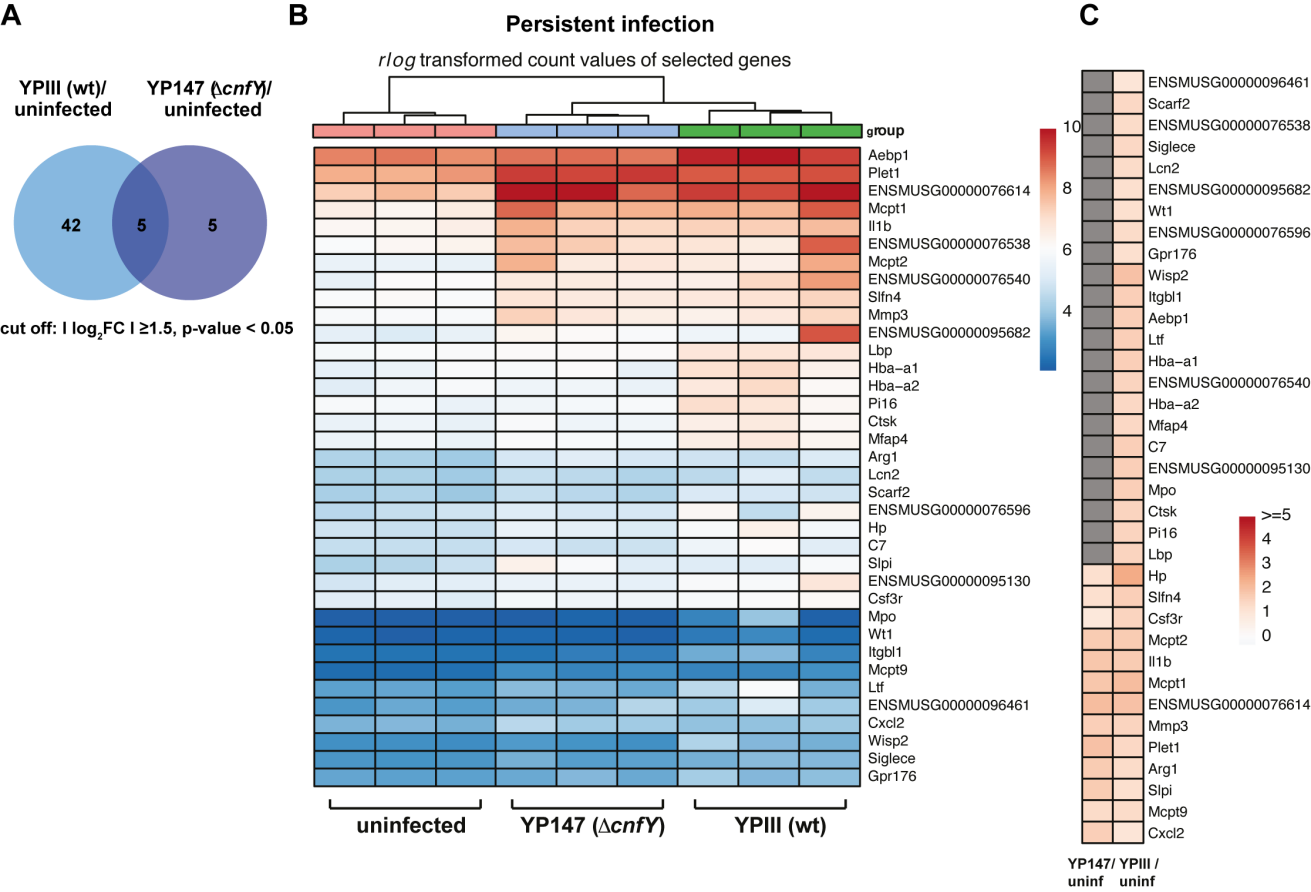
The host transcriptome during persistent *Yersinia* infection in the presence and absence of the CNF_Y_ toxin. (**A**) Venn-diagram of differential expressed genes (cut off: Ilog_2_FCI≥1.5) from uninfected at 42 dpi versus YPIII or YP147(Δ*cnfY*) at 42 dpi. (**B**) Heat map of top enriched (red) and depleted (blue) host transcripts based on *DESeq*2 analyses. Color-coding is based on *rlog* transformed read count values. (**C**) Heat map illustrates log2 fold changes of host transcripts detected in YPIII- or YP147(Δ*cnfY*)-infected mice compared to uninfected mice (adjusted P value ≤ 0.05). Grey boxes: not significant.

In contrast to the acute phase, a **|**log_2_FC**|** ≥ 1.5 of host transcript levels was observed for only 47 transcripts in the wildtype-infected cecal tissue and for 10 of the transcripts in the Δ*cnfY* mutant-infected tissue (Fig **6A**, **S7B** Fig). A comparative analysis of host transcript changes illustrated that in particular the proinflammatory responses are down-regulated (Hp, Cxcl2, IL-1α, Mmp3/8, Clec4d/e) or fully eliminated (e.g. IL-6, Saa3) when the bacteria enter the persistent stage. It further demonstrated that bacterial loads >10^4^ CFU/g cecum at 42 dpi are tolerated without an explicit change of the host transcriptome (Fig **6B and 6C**, **S4**–**S6** Datasets). Only a few transcripts were commonly enriched during persistence of both strains, e.g. IL-1β, mast cell proteases (Mcpt1,2,9), tissue repair (Plet1, Aebp1) and the immune modulator arginase 1 (Arg1) (Fig **6B and 6C**, **S4** **and** **S5** Datasets). Arg1 depletes arginine in the tissue environment and thereby impairs NO production and T cell immunity by inhibiting T cell proliferation, memory and T cell receptor expression [28]. A higher transcript abundance was also observed for the secretory leukocyte protease inhibitor (Slpi), which inhibits net formation [43], the neutrophil-specific protein Slfn4, and the chemokine Cxcl2, altogether indicating that neutrophils and mast cells are recruited during *Yersinia*-persistence. The majority of other enriched host transcripts important to counteract the pathogen (e.g. ion chelators Hp, Ltf, Lcn2; complement factor C7; pathogen recognition protein Lbp, Mmps, and immune modulator Wisp2/CCN5) were not or less enriched in Δ*cnfY*-infected ceca (Fig **6B and 6C**; **S4**–**S6** Datasets). In summary, our data show that although host-generated responses towards a persistent *Y*. *pseudotuberculosis* wildtype infection is already very restricted, the persistent infection is even further unrecognizable by the immune system in the absence of CNF_Y_.

### CNF_Y_ deficiency impacts cytokine responses

**Fig 7:**
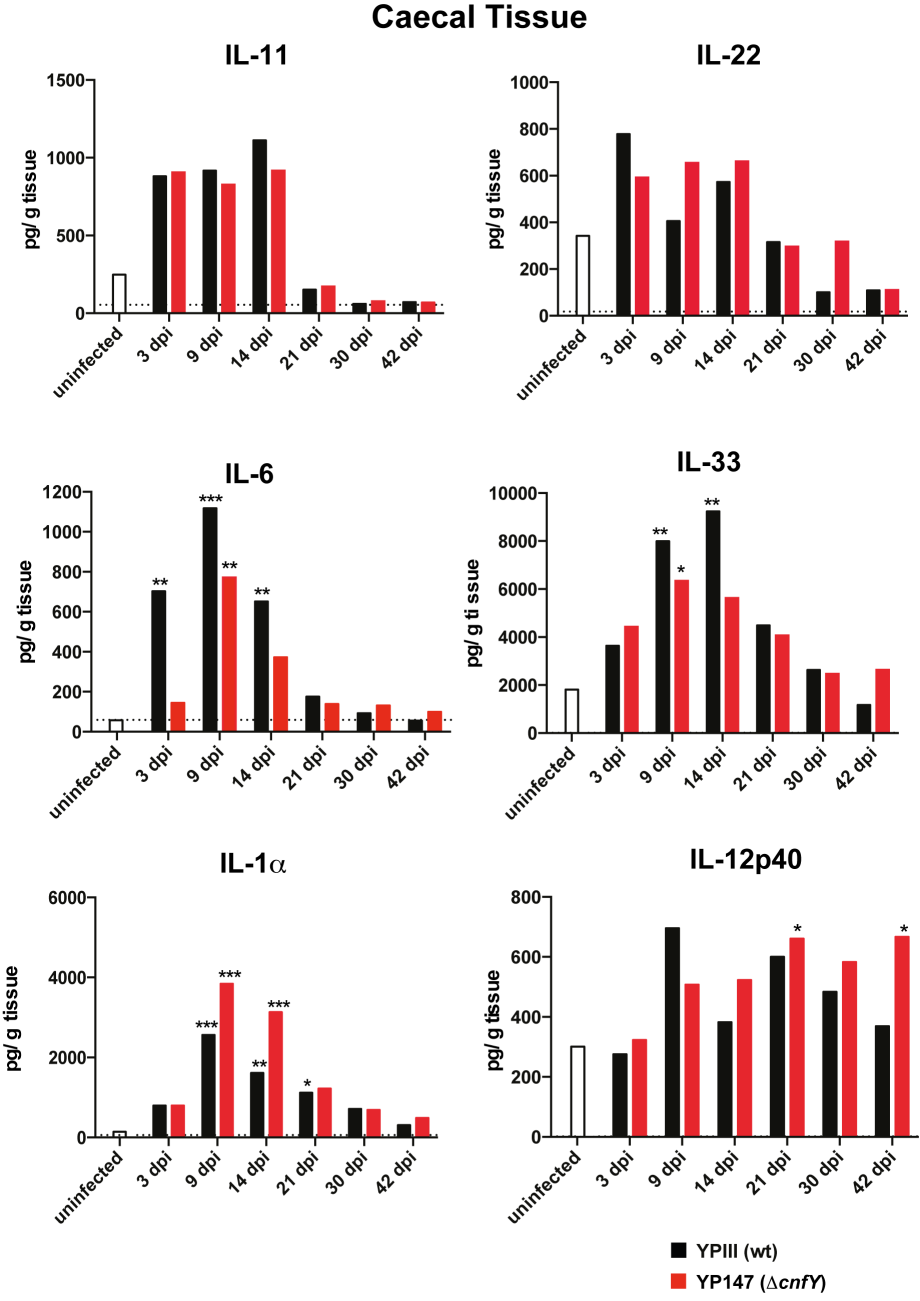
Cytokine responses in wildtype- and Δ*cnfY* mutant-infected cecal tissue. BALB/c mice were orally infected with 10^6^ CFU of YPIII or YP147(Δ*cnfY*). At indicated time points post infection, the cytokines in the cecal tissue were determined. The bars represent the geometric mean of three independent experiments using n = 3–9 mice/group and the dotted line illustrates the detection limits. The cytokine level at any given time point between wildtype- and Δ*cnfY* mutant-infected mice was analyzed with the Kruskal-Wallis test and Dunn's correction, P-values: * <0.05, ** <0.01, *** <0.001.

The present study revealed that distinctive immune and inflammatory responses are detectable in the histopathological and the host transcriptome analyses during infection with the *Y*. *pseudotuberculosis* wildtype strain YPIII and its isogenic *cnfY* mutant derivative (Figs **2, 5 and 6**). We therefore analyzed whether this is also reflected in an altered cytokine response in the cecal tissue of infected mice using a multiplex assay. In agreement with the transcriptomic data, several cytokines (e.g. IL-11 and IL-22) were found to increase upon infection, but no strong difference was observed between wildtype and Δ*cnfY* mutant-infected animals (Fig **7**). Among the few cytokines that were differentially produced was the pleiotrophic cytokine IL-6, which was more rapidly and strongly increased in wildtype- compared to Δ*cnfY* mutant-infected cecal tissue (Fig **7**). This also supports RNA-seq results, showing that IL-6 transcript levels are significantly reduced in the absence of CNF_Y_ (Fig **5**, **S1–S3** Datasets). In addition, IL-33, an alarmin released upon barrier disruption, was slightly more induced during wildtype infection. As IL-6 induces acute phase responses and attracts neutrophils to the infection sites [44], and IL-33 participates in pathological fibrotic reactions and promotes responses by cytotoxic NK cells and CD8^+^ T cells during microbial invasion [45], it is likely that they contribute to increased inflammation and tissue damage in wildtype-infected ceca. Moreover, levels of almost all tested cytokines are downregulated during development of persistency, indicating a dampened immune response.

In contrast, IL-1α levels were higher in Δ*cnfY* mutant-infected ceca during early acute phase, and also the amount of the IL-12p70/IL-23 subunit IL-12p40 was increased during later infection stages compared to wildtype-infected ceca (Fig **7**). IL-1α is a passively released danger signal from dying cells, which provokes neutrophil, CD8^+^ T cells and T_reg_ recruitment, and activates inflammatory mediators [46]. IL-12 activates T cell differentiation, production of IFN-γ by CD8^+^ T cells and NK cells, and regulates the Fe^2+^/Zn^2+^ content in the tissue [47]. This supports previous data (**S1–S3** Datasets), demonstrating that major proinflammatory cytokines are still induced by both, the wildtype and the Δ*cnfY* mutant strain, although the overall inflammatory pathology of the *cnfY* mutant-infected ceca is significantly reduced (Fig **2**).

The precise mechanisms that dampen the inflammation and allow establishment of *Yersinia*-persistence are still unclear. However, the overall impact of CNF_Y_ suggests a set of immune responses (e.g. differential levels of IL-6, immune modulators, bacteriocidal activities and anti-inflammatory factors such as Tnip3, **S1–S3** Datasets), which may contribute to this process.

### Absence of CNF_Y_ promotes early remodeling of *Yersinia* gene expression

**Fig 8:**
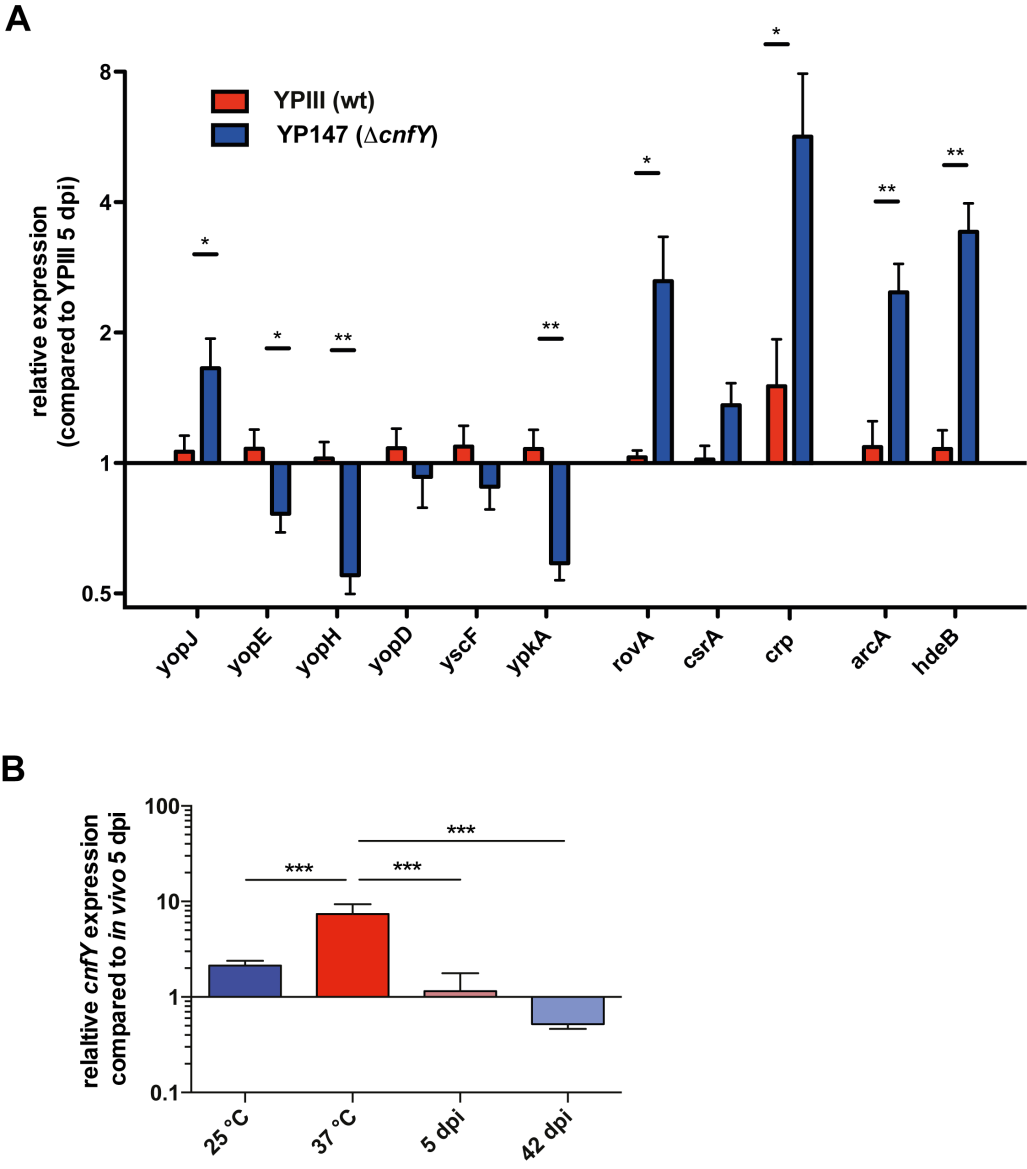
Expression pattern of *Yersinia* persistence genes. (**A**) Relative changes in transcript abundance of selected *Yersinia* genes in YPIII- or YP147 (Δ*cnfY*)-infected ceca 5 dpi. qRT-PCR was performed with total RNA from Tissue RNA-seq samples. (**B**) Relative expression of *cnfY* from total RNA from YPIII- or YP147 (Δ*cnfY*) grown *in vitro* at 25°C and 37°C, and isolated from infected ceca 5 and 42 dpi. The data show the mean +/- SEM of at least three independent experiments performed with at least two technical replicates and were analyzed by multiple t-tests employing Holm-Šídák’s correction, P-value: * <0.05, ** <0.01.

Avican *et al*. [10] demonstrated that persistent *Y*. *pseudotuberculosis* undergoes transcriptional reprogramming when the bacteria reside in the cecal tissue. To identify mechanism that enhance establishment of *Y*. *pseudotuberculosis* persistence in the absence of CNF_Y_, we also analyzed the gene expression profiles of the bacteria in the cecum during acute and persistent infection by our tissue dual RNA-seq approach. However, the recovered total RNA of the 10^4^−10^6^ CFU from the cecal tissue was not sufficient to obtain full transcriptome coverage due to the very low abundance of unique *Y*. *pseudotuberculosis* transcripts. We therefore decided to test relative mRNA abundance of selected genes, which were previously shown to be reprogrammed during the transition from the acute to the persistent stage [10]. To cover the most affected metabolic and physiological functions, we first investigated transcript abundance of different anaerobiosis and stress adaptation genes by qRT-PCR. A selection is shown in **S8A Fig** In agreement with previous results [10], the genes were upregulated during the persistent phase. Moreover, no difference was detectable between the wildtype and the *cnfY* mutant, indicating that adaptation during persistency is similar (**S8A** Fig). This is in contrast to the acute phase, in which this set of genes undergoes a pre-early reprogramming in the Δ*cnfY* mutant towards the persistent mode. For instance, transcripts enriched under persistence (e.g. *arcA* for anaerobic metabolism, and *hdeB* for acidic stress) are already more abundant during the acute phase (Fig **8A**, **S8A** Fig); yet, transcript levels do not vary between wildtype and the mutant during growth *in vitro* (**S8B** Fig). This suggests that these genes undergo a pre-early reprogramming in the Δ*cnfY* mutant towards the persistent mode.

This observation prompted us to test whether also important virulence traits, which are differentially expressed during persistence [10], underwent pre-early reprogramming in the Δ*cnfY* mutant. In fact, we found that the expression of the global virulence regulator genes (*csrA*, *crp*, *rovA*) controlling adhesion/invasion factors and motility important for the early stages of the infection were more upregulated at 5 dpi in the *cnfY* mutant compared to the wildtype. In contrast, genes encoding the T3SS needle component YscF and the secreted effectors YopE, YopH, YopD and YpkA were downregulated in the *cnfY* mutant strain during the acute phase (Fig **8A**), very similar to the development of a persistent infection by the wildtype [10]. These data support our previous assumption that elimination of CNF_Y_ seems to trigger a pre-early reprogramming towards the persistence program. However, in contrast to the other T3SS/*yop* transcripts, the mRNA of effector YopJ, which dampens innate immune responses and modulates inflammasome signaling [48], was enriched in the *cnfY* mutant (Fig **8A**). This suggests that *yopJ* expression is decoupled and does not follow preterm reprogramming.

Moreover, we found that expression of *cnfY* is significantly lower during infection, in particular during the persistent stage, compared to growth *in vitro* (Fig **8B**). This indicated that absence or downregulation of CNF_Y_ might provoke a different host response in the cecal tissue during the early stages of the infection that drives wildtype gene expression into the persistent mode.

## Discussion

Several enteric pathogens, including yersiniae are able to persist in the intestinal tract and associated lymphatic tissues and can promote the development of chronic arthritis and ileitis [6]. The basis for this process is formed during the acute infection phase. It is determined by the complex pathogen-triggered immune reactions, yet the participating molecular players of the pathogen are largely unknown. In this work, we demonstrate that removal of a single virulence factor, the CNF_Y_ toxin, is sufficient to dampen inflammation and to evade the host’s immune defense favoring establishment of *Yersinia*-persistence.

**Fig 9:**
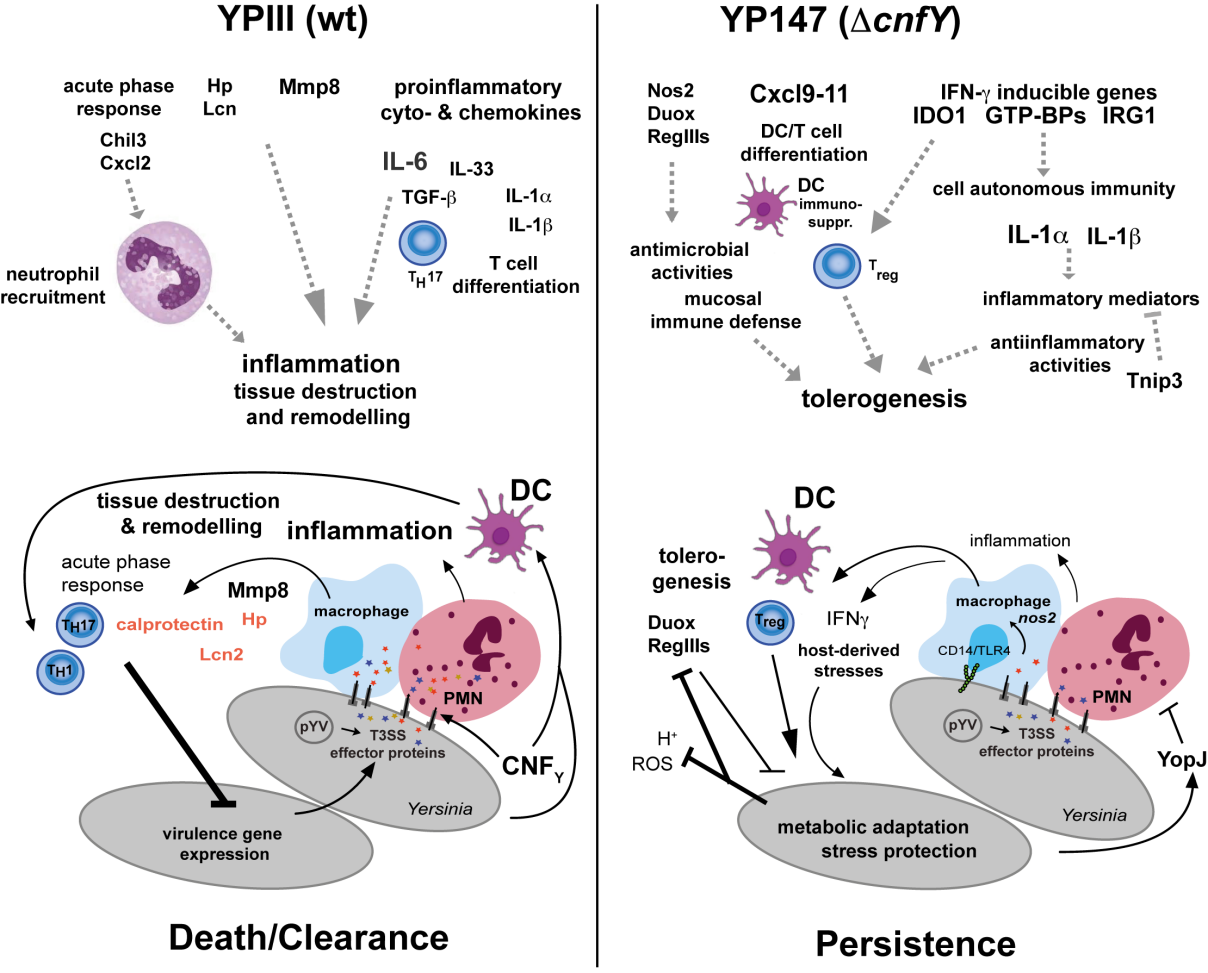
Model of CNF_Y_ influence on the development of *Yersinia* persistence. Schematic overview of induced inflammatory and acute phase responses, which are triggered by the *Y*. *pseudotuberculosis* strain YPIII and the isogenic CNF_Y_-negative variant based on the transcriptome analysis of *Yersinia*-infected cecal tissue 5 dpi. Host responses that are expected to result from altered inflammatory responses and other defense reactions, are indicated by dashed arrows. Transcript-based adaptations of *Y*. *pseudotuberculosis* and the host during colonization of the cecum during acute and persistent infection and their influence on the outcome of the infection are illustrated.

Here we show, that the CNF_Y_-triggered process represents a double-edged sword. On the one hand, CNF_Y_ presence increases inflammation and IL-6/IL-33 levels, which promotes (i) acute phase responses, (ii) induces coagglutination, and (iii) enforces neutrophil recruitment leading to a higher production of reactive oxygen species and proteases [44]. This process significantly contributes to the exacerbated bacteria-induced inflammatory response, massive tissue damage and dysbiosis, resulting in rapid death or elimination of the infection in the majority of infected animals (Fig **9**). On the other hand, CNF_Y_-induced inflammation might facilitate systemic spread. Moreover, inflammatory mediators such as IL-6 and TGF-β are known to induce the development of T_H_17 cells and inhibit differentiation of regulatory T cells (T_reg_) [44], which may also promote protection against extracellular bacterial infections. To test whether the reduction of IL-6 promoted inflammation plays a major role in the establishment of *Yersinia* persistence, we further tried to deplete IL-6 by antibodies as described [49–51]. In all previous studies, the antibody was successfully used to deplete IL-6 from non-infected mice, but IL-6 depletion from *Yersinia*-infected mice failed.

In contrast, acute infection with the Δ*cnfY* mutant is characterized by the induction of multiple IFN-γ dependent genes (e.g. antigen presentation, Nos2 and GBPs) that destabilize bacterial-containing vacuoles and promote cytosolic LPS release [52]. Cytosolic LPS triggers phagocyte pyroptosis via caspase-11 activation and initiates release of IL-1α [53, 54], a proinflammatory cytokine, which is more strongly produced in the Δ*cnfY* mutant compared to wildtype-infected mice. This is accompanied by increased expression of numerous bactericidal activities, which avoid systemic inflammation, prevent alteration of the symbiotic microbiota, and decrease disease severity (Fig **9**). We further observed significant higher transcript levels of the IFN-γ inducible Ido1, a multifaceted enzyme of the tryptophan catabolism, promoting immune tolerance [35]. This suggests that Ido1-induced tolerogenesis may play a role in protecting the Δ*cnfY* mutant from deleterious attacks by the immune system, which could support the shift into persistence. Tryptophan depletion via Ido1 was first described as a mechanism to inhibit growth of intracellular pathogens, but it is also reported to induce persistence of intracellular bacteria (e.g. *Chlamydia pneumoniae*) and to contribute to chronic disease [55, 56].

Another emerging concept is that the onset of inflammatory reactions due to invading bacteria, including *Yersinia* provokes an alteration of the symbiotic microbial community in the intestinal compartment [10, 17, 57]. Here, we show that a CNF_Y_-positive *Y*. *pseudotuberculosis* wildtype strain causes an outgrowth of Proteobacteria and a massive decrease of Bacteroides. A severe consequence of sustained microbiota-induced inflammation and tissue remodeling can be the disruption of the communication between the immune system and the tissue, which persistently compromises tissue immunity and homeostasis, a phenomenon named 'immunological scarring' [16]. It is unclear which of the observed changes of immune reactions or microbial communities are a cause or a result of the pathogen-induced inflammatory responses. However, it is most likely that a combination of the immune-status of the host and metabolic signals of the environment influence the outcome of the infection, i.e. bacterial clearance or persistency [58]. Specifically, work by DePaolo and colleagues [17] showed that elevated levels of reactive oxygen species produced by recruited neutrophils upon a *Y*. *enterocolitica* infection reduce commensal-produced thiosulfate to tetrathionate. Tetrathionate can be utilized as respiratory electron receptor by certain δ-Proteobacteria, including *Y*. *pseudotuberculosis*, which promotes their outgrowth. As observed shift of microbiota is prohibited in the absence of CNF_Y_, we assume that Δ*cnfY* mutant-driven inflammation is too mild to produce sufficient amounts of tetrathionate that allows δ-Proteobacteria expansion and induction of dysbiosis. In addition, upregulation of other factors, e.g. fucosyltransferase 2 (Fut2), which delivers fucosylated metabolites to the gut microbiota as defense against invading bacteria [59, 60], could support its compositional stability.

This work further shows that persistence of *Yersinia* is characterized by a dampened immune response. Presence of the Δ*cnfY* mutant is nearly inapparent, and only a faint host response was detectable against wildtype bacteria, which includes immune suppression mechanisms, such as the arginine-depleting enzyme Arg1. Arg1 controls T cell activation and proliferation, and was found to suppress inflammation and tissue damage during the persistent stage of several intracellular viral and bacterial pathogens, e.g. *Mycobacterium tuberculosis* [61].

Another important aspect in the development of persistence is that elimination of CNF_Y_ is enough to cause a pre-early reprogramming of *Y*. *pseudotuberculosis* to its persistence program. How pre-early reprogramming is initiated is still unclear, but it is highly likely that the induction of a differential immune response provokes a different adjustment of the bacterial expression profile (Fig **9**). This includes the pre-adaptation of the bacterial metabolism for anaerobic growth and an improvement of the overall stress resistance of the pathogen. Moreover, multiple important virulence traits are differentially regulated, which permit long-term colonization of the tissue, but in parallel keep the immune system at bay.

Presence of the CNF_Y_ toxin strongly enhances the activation of small Rho GTPases and the delivery of anti-phagocytotic and apoptotic Yop effector proteins into immune cells [12, 13]. Thus, it is tempting to speculate that a reduction of these processes in the absence of CNF_Y_ decreases inflammation and enhances the establishment of a persistent infection. The Rho GTPases and their immediate downstream effectors are key regulators of cellular actinomyosin dynamics and as such crucial for leukocyte motility [62, 63]. Consequently, absence of CNF_Y_ would reduce tissue infiltration of leukocytes and inflammation as seen in ceca of *cnfY* mutant-infected mice. Moreover, upregulation of YopJ, which dampens TLR-induced expression of proinflammatory cytokines by interference with the MAPK and NF_k_B pathways [64, 65], and caspase-1 promoted IL-1β production [66], could overcome low translocation activity in the absence of CNF_Y_ and help to dampen inflammation during persistence. However, this process is also accompanied by a decreased expression of other effectors such as YopE, YopH, YopM and YpkA [10, 11] (Fig **8A**). These effectors were found to inhibit Rho GTPases and/or perturb host immune responses, including the production of certain pro-inflammatory cytokines, the maturation of caspases and the activation of the inflammasome [67–69]. At this time, it is still unclear how CNF_Y_-mediated changes of (i) individual Rho GTPases in targeted immune cells, (ii) the Yop translocation efficiency and (iii) the expression of the individual T3SS/Yop components influence inflammation and development of a persistent infection. As the different components are all part of a highly complex network, a more detailed analysis of their interplay and outcome of their actions during the acute and the persistent infection stage is necessary to dissect the contribution of the individual factors. Nonetheless, importance to suppress exacerbated inflammation to drive bacterial pathogens into persistence is substantiated by the fact that also other chronic pathogens such as *Helicobacter pylori* [70] use distinct strategies to avoid induction of inflammation and immune recognition (e.g. by modification of lipid A).

Many clinical *Y*. *pseudotuberculosis* isolates harbor deletions within the *cnfY* gene [71]. Based on the results of this study, this loss does not only enhance long-term persistency, it also confers continuous shedding of the pathogen into the environment, which facilitates transmission to other host reservoirs. The homologous toxin CNF-1 of *E*. *coli* is also only present in less than 36–48% of uroseptic human isolates [72, 73]. The role of CNF-1 for *E*. *coli* virulence is still not clear, but a recent study demonstrated that CNF-1 activity decreases the pathogen load by potentiating LPS-triggered IL-1β-mediated antimicrobial host responses, but favors survival during bacteremia [74]. One reason for this diversity may be that the individual virulence factor armamentarium or expression pattern of certain strains may relieve the pressure to retain the CNF toxin, e.g. sufficient expression of the Yop/T3SS in *Yersinia*. In the opposite, CNF-promoted tissue damage accelerates pathogen access to deeper tissues and facilitates establishment of systemic infections, a property beneficial to strains with reduced tissue invasion properties.

Taken together, our study indicate a tight balance between (i) *Yersinia*-triggered inflammation and death/clearance mechanisms, and (ii) *Yersinia*-induced immune suppression and tolerance allowing its long-term persistence. We further discovered that modulation of a single bacterial factor, the secreted toxin CNF_Y_, is sufficient to shift this balance and change the fate of a *Y*. *pseudotuberculosis* infection. A more in-depth analysis of the individual identified host responses throughout the course of infection will give valuable information for the design of better ways to evaluate, treat and prevent persistent infections.

## Material and methods

### Bacterial strains, cell culture, media and growth conditions

The strains used in this study are listed in **S3** Table. Overnight cultures of *E*. *coli* were routinely grown at 37°C, *Yersinia* strains were grown at 25°C or 37°C in LB (Luria-Bertani) broth. The antibiotics used for bacterial selection were as follows: carbenicillin 100 **μ**g/ml, and kanamycin 50 **μ**g/ml.

### DNA manipulations, construction of plasmids and strains

All DNA manipulations, PCR, restriction digestions, ligations and transformations were performed using standard techniques as described previously [75, 76]. Plasmids used in this study are listed in **S3** Table.

The mobilizable suicide plasmid pWH9 was constructed to integrate the *mRuby2* gene under the control of the constitutive *LtetO-1* promoter (*P*_*LtetO-1*_::*mRuby2*) into the intergenic region between the locus YPK_3294 and YPK_3295 at position 3606960–3607832 (NCBI accession path >gi/170022262/ref/NC_010465.1/:3606960–3607832). For this purpose, two chromosomal fragments upstream and downstream of the integration site were amplified by PCR from genomic DNA of *Y*. *pseudotuberculosis* YPIII with primer pairs VI392/VI394 and VI393/VI395. Subsequently, a combined fragment which created a *Xho*I and *Not*I cloning site at the fusion site was amplified by PCR using primer pair VI393/VI395 and both fragments as templates. The generated fragment was integrated into the *Sac*I site of plasmid pAKH3. The resulting plasmid pWH9 and a fragment encoding *P*_*LtetO-1*_::*mRuby2* were both digested with *Not*I and *Xho*I and ligated, generating plasmid pWH14. For the *P*_*LtetO-1*_::*mRuby2*-encoding fragment, the LtetO-1 promoter and the ribosome binding site from plasmid pFS43 was amplified by PCR using primers VI545/VI556. The *mRuby2* gene, kindly provided by M. Erhardt as synthetic gene fragment based on vectors of [77], was amplified by PCR with primer pairs VI557/VI548. The resulting fragments were fused by PCR using primer pairs VI545/VI548. The sequence of pWH14 was verified by sequencing with primers III981, III982, VI392 and VI395.

The *mRuby2* gene, encoding the red fluorescent protein mRuby2, was cloned under the control of the tetracycline promoter and inserted into the intergenic region between YPK_3294 (LysR-type transcriptional regulator) and YPK_3295 (aminoacyl-histidine dipeptidase *pepD*) cloned onto suicide plasmid pWH9. This locus of *Y*. *pseudotuberculosis* is transcriptionally silent under various tested *in vitro* growth conditions and within mouse Peyer’s patches as verified by RNA-Seq analyses [22, 78]. Red-fluorescent *Y*. *pseudotuberculosis* strains YP339 (YPIII *P*_*LtetO-1*_::*mRuby*) and YP340 (YPIII Δ*cnfY*, *P*_*LtetO-1*_::*mRuby*) were obtained by the integration of the generated suicide plasmid pWH14 via conjugation into the *Y*. *pseudotuberculosis* YPIII genome as described earlier [79, 80]. To obtain derivatives of the conjugates, which spontaneously lost the integrated plasmid, including the *sacB* and *bla* resistance gene, but maintained the *P*_*LtetO-1*_::*mRuby2* gene, fast growing and carbenicillin-sensitive bacteria were selected on 10% sucrose plates. The correct chromosomal insertion of P_L*tet*O-1_::*mRuby2* into the *Yersinia* chromosome was tested by PCR and sequencing with primers VI504, VI505, VI545, VI548 (**S3** Table) and expression of the red fluorescent mRuby2 proteins was evaluated by fluorescence microscopy.

### Ethics statement

All experiments were performed in strict accordance with the German Recommendation of the Society for Laboratory Animal Science (GV-SOLAS) and the European Health Recommendations of the Federation of Laboratory Animal Science Associations. The animal protocol was approved by the “Niedersächsisches Landesamt für Verbraucherschutz und Lebensmittelsicherheit”: (33.9-42502-04-13/1166). Mice were housed under specific pathogen-free conditions with free access to food and water. Mice were allowed to acclimate to the new housing conditions for one week prior to the infection, and every effort was made to reduce suffering.

### Mouse infection

7 week-old female BALB/c mice were purchased from Janvier (Saint Berthevin Cedex, France). To monitor acute and persistent *Y*. *pseudotuberculosis* infections, groups of 5–20 animals were orally infected with approximately 10^6^−10^8^ bacteria of *Y*. *pseudotuberculosis* strains YPIII and YP147 (Δ*cnfY*) using a gavage needle. Bacteria used for the infection experiments were grown over night in LB medium at 25°C, washed and resuspended in PBS. The infected mice were monitored every day for the first 14 days post infection and subsequently twice a week for 42 days (1x10^6^ CFU) to determine survival, health status and body weight. For the assessment of the bacterial loads in the feces, feces were sampled from individual living mice at specific time points, weighed and homogenized in BHI containing 1 μg/ml irgasan. For the analysis of the bacterial load in the cecum, mice were euthanized by CO_2_ asphyxiation at specific time points after infection. The organ contents were collected by flushing of the cecum with 10 ml sterile 1 x PBS. The ceca were weighed and homogenized in PBS at 22.000 rpm for 10 sec using a Polytron PT 2100 homogenizer (Kinematica, Switzerland). To determine the bacterial load of the feces and the cecum, serial dilutions of the homogenates were plated on LB plates with 0.5 μg/ml irgasan. The colony forming units (cfu) were counted and are given as cfu per g organ/tissue. To determine whether the feces contain *Yersiniae*, the remaining homogenate of the feces was incubated in BHI medium supplemented with 0.5 μg/ml irgasan and incubated over night at 25°C with shaking.

### Analysis of mRuby2-expressing *Y*. *pseudotuberculosis* strains

To exclude influence of *mRuby2* expression on *Yersinia* pathogenesis we characterized growth and virulence of the isogenic *mRuby2*-expressing *Yersinia* strains (YPIII *mRuby2*, YPIII *mRuby2* Δ*cnfY*). We found that neither *in vitro* growth (**S2A** Fig) nor virulence (**S2B–S2E** Fig), documented by mouse survival, weight loss of the infected animals, and bacterial numbers in the cecum, was affected by mRuby2 expression. Moreover, we did not observe any changes in the development of persistent infection and the colonization efficiency in the acute and persistent mode (**S2F and S2G** Fig). This demonstrated that the fluorescent strains are suitable for *in vivo* localization studies. For the analysis of the bacterial colonization patterns by fluorescence microscopy, whole single channel microscopic pictures (DAPI, mRuby2) were adjusted for exposure and brightness. Detail views were cropped from the overview picture and not manipulated further. The size of the microcolonies were scored in fields of multiple sections (approximately 15–20 sections per mouse, 3 mice in total) according to the following scoring criteria: 0: no microcolonies in lymphoid tissue section (diameter < 20 μm), 1: smaller-size microcolony (diameter < 50 μm), 2: larger-size microcolony (diameter > 100 μm).

### Histology

For hematoxylin and eosin (H & E) staining, ceca of mice positively tested for *Yersinia* were excised at the indicated time points, fixed in 4% formalin for 24 to 48 h and embedded in paraffin. 3 μm sections were stained with H & E. For each group 3 to 5 mice were blindly analyzed with light-microscopy by a histopathologist (see **S1** Fig).

To localize bacteria and analyze the bacterial colonization pattern in the infected tissue, mice infected with fluorescently-labeled *Y*. *pseudotuberculosis* YPIII (*mRuby2*) or YPIII Δ*cnfY* (*mRuby2*) were sacrificed by CO_2_ asphyxiation at day 3 or day 42 post infection. The cecum was isolated and cryosections of the ceca were prepared for fluorescent microscopy as described [81]. The cryo-sections were examined with the Axiovert II fluorescence microscope (Zeiss) using the Axiocam HR digital CCD camera (Zeiss) and the software ZEN 2012 (Zeiss). Obtained images were further processed using Adobe Photoshop CS4 (version 11.0; Adobe Systems Incorporated).

### Chemokine and cytokine profiling

To determine chemokine and cytokine concentrations in the serum and tissues of untreated and *Y*. *pseudotuberculosis* infected mice, tissue samples were taken and subjected to a multiplex immunoassay. The global cytokine profiles, of tissue lysates from ceca isolated from uninfected and infected mice 3, 9, 14, 21, 30 and 42 days post infection was determined with LEGENDplex bead-based immunoassays “T helper cytokine panel” and “Cytokine Panel 2” (BioLegend, www.biolegend.com). For this purpose, isolated ceca were flushed with 1 x PBS, weighed and snap-frozen in liquid nitrogen. The cecal contents were plated in serial dilutions to determine the *Y*. *pseudotuberculosis* load. For protein isolation, frozen ceca were added to 2 ml ice-cold NP-40 buffer (150 mM NaCl, 50 mM Tris-HCl (pH 8.0), 1% NP-40, 1 mM PMSF), were homogenized (15,000 rpm, Polytron PT 2100) on ice and large debris was pelleted by centrifugation (4°C, 1,000 g, 5 min) and discarded. Protein concentrations were determined with the Pierce BCA protein assay kit (ThermoFisher Scientific) according to the manufacturer’s recommendations. The LEGENDplex assay was performed according to the manufacturer’s instructions and the beads were detected with the flow cytometer (LSRFortessa, BD Biosciences) following the assay requirements for flow cytometer setups and acquisition. Data analysis was performed with the LEGENDplex Data Analysis Software V7.0 (BioLegend).

### Microbial community (16S DNA-sequencing)

To compare the microbial composition of mice when the infection switches from the acute to the persistent stage 16S rDNA sequencing was performed of the bacterial community of the feces. For this purpose 10 to 70 mg feces were directly sampled from individual mice. The feces were weighed, and homogenized in 1 ml BHI medium. 100 μl was for serial dilutions and plated on LB plates with 0.5 μg/ml irgasan to assay the *Y*. *pseudotuberculosis* loads. The remaining homogenate was pelleted, and frozen at -20°C. The DNA of the microbial community was extracted by a combined method using mechanical disruption (bead-beating) and phenol/chloroform-based purification [82]. Frozen feces samples were suspended in a solution containing 500 μl of extraction buffer (200 mM Tris, 20 mM EDTA, 200 mM NaCl, pH 8.0), 200 μl of 20% SDS, 500 μl of phenol:chloroform:isoamyl alcohol (24:24:1) and 500 μl of 0.1 mm zirconia/silica on ice. Samples were homogenized twice with a bead beater (BioSpec with 0.1 mm beats) for 2 min and centrifuged (3 min, 8.000 rpm, 4°C). The supernatant was transferred into one vol of phenol:chloroform:isoamyl alcohol (24:24:1), centrifuged (3 min, 8.000 rpm, 4°C) and the supernatant was precipitated with 0.1 vol 3 M sodium acetate (pH5.5) and 1 vol isopropanol. The precipitate was pelleted by centrifugation (20 min, 13.000 rpm, 4°C), washed with 70% ethanol, dried with a Speedvac and resuspended in 200 μl TE buffer with 100 μg/ml RNase A (2 min, RT). The DNA was purified using spin columns (BioBasic) according to the manufacturer's instructions and adjusted to 25 ng/μl.

Amplification of the V4 region (F515/R806) of the 16S rRNA gene was performed in triplicates using Q5 DNA polymerase and barcoded primers for 16S DNA as described previously [83]. Triplicates were pooled, quantified using PicoGreen and adjusted to 10 mM. The 16S libraries were quantified with KAPA Library Quantify KIT and sequenced on an Illumina MiSeq platform (PE250). Filtering of sequences for low quality reads and barcode-based binning was performed using QIIME v1.8.0 [83]. Reads were clustered into 97% ID OTUs using UCLUST, followed by taxonomic classification using the RDP Classifier executed at 80% bootstrap confidence cut off [84, 85]. Sequences without matching reference dataset, were grouped as *de novo* using UCLUST. Phylogenetic relationships between OTUs are determined using FASTTREE to the PyNAST alignment [86]. The OTU absolute abundance table and mapping file are used for statistical analyses and data visualization in the R statistical programming environment package PHYLOSEQ [87].

### Total RNA extraction from bacterial cultures and mouse tissue

To assess the expression patterns of selected bacterial genes by qRT-PCR, RNA of bacterial *in vitro* cultures was isolated. For this purpose, *Y*. *pseudotuberculosis* was grown at 25°C or 37°C for 16 h in triplicates. The cultures were pooled, pelleted by centrifugation (14.000 g; 4°C; 2 min), resuspended in 0.2 volumes stop solution (5% (v/v) water-saturated phenol in ethanol) and snap-frozen in liquid nitrogen. The suspension was thawed on ice and centrifuged (14.000 g; 4°C; 2 min). Subsequently, the pellet was resuspended in lysozyme-TE buffer (50 mg/ml) and incubated for 10 min at room temperature. The RNA of the sample was purified with the SV Total RNA-Isolation kit (Promega) following the manufacturer’s instructions. The RNA was eluted into a reaction tube in 100–200 μl RNase-free water and used for qRT-PCR.

For the preparation of total RNA of murine ceca, uninfected equally aged mice and mice infected with either *Y*. *pseudotuberculosis* YPIII or YPIII Δ*cnfY* were sacrificed at 5 or 42 dpi. Infection doses were adjusted (10^6^−10^8^ CFUs) to obtain an equal colonization during the acute and persistent stage of the infection. The ceca of uninfected or colonized mice were removed, extensively flushed with 1 x PBS and snap frozen in liquid nitrogen. The contents of the flushed ceca were analyzed for *Y*. *pseudotuberculosis* loads as described above to determine the severity of colonization. For total RNA isolation, snap frozen ceca were added to 4 ml freshly prepared lysis solution (4 M guanidinium thiocyanate, 25 mM sodium citrate, 0.5% N-laurosylsarcosine (w/v), 0.1 M β-mercaptoethanol) [88] and homogenized on ice at 11.000 rpm (Polytron PT2100, Kinematica) for 10 s. Total RNA of the homogenates was purified as described [22]. The quality of the total RNA extracts was analyzed with the Agilent 2100 Bioanalyser (Agilent Technologies). To obtain three independent pooled replicates, RNA extracts isolated of ceca from 3 to 5 mice per group were pooled to one of three replicates. To remove contaminating DNA traces, total RNA extracts and pools were treated with TURBO DNase (Ambion) following the manufacturer’s specifications.

### Depletion of mouse rRNA and Illumina ScriptSeq library preparation

For mouse rRNA depletion and RNA library preparation of total RNA pools human/mouse/rat ScriptSeq complete kit (Illumina) was employed according to the manufacturer’s instructions, but with the following change. For this purpose, 1 μg of DNA-depleted total RNA was depleted for murine rRNA with the Ribo-Zero kit (human/mouse/rat) following the manufacturer’s specifications. After rRNA depletion External RNA Controls Consortium (ERCC) spike in control mixes 1 or 2 (Ambion) were added to determine the dynamic range, lower detection limit and accuracy of differential gene expression measures. The quality of the libraries was validated using Agilent 2100 Bioanalyzer (Agilent Technologies) following the manufacturer’s advice.

### RNA sequencing, bioinformatic processing and overrepresentation and pathway analyses

The Single-end strand specific sequencing of the finalized libraries (short reads) was performed with the HiSeq2000. The obtained data was processed as described [22, 78]. All libraries have been assessed for sufficient read quality and potential contamination using the *FastQC* program (http://www.bioinformatics.babraham.ac.uk/projects/fastqc/). The quality assessment showed neither insufficient read quality, nor nucleotide frequency biases introduced by primer contamination. Therefore, libraries were directly aligned to the mouse genome (assembly: GRCm38/mm10) using the splice junction mapper *TopHat2* [89] with library type *fr-secondstrand*. Reads aligned to annotated genes were quantified with the *htseq-count* program [90] using gene annotations from Ensembl release 75. Determined read counts served as input to *DESeq2* [21] for pairwise detection and quantification of differential gene expression. For DESeq2 parametrization we used a beta prior and disabled the Cook distance cut off filtering. All other parameters remained unchanged. In addition, RPKM (reads per kilobase max. transcript length per million mapped reads) values were computed for each library from the raw gene counts. The list of *DESeq2* determined differentially expressed genes (DEGs) was filtered with an absolute log_2_ fold change cut-off of at least 1.5 and a cut-off for a multiple testing corrected p-value of at most 0.05. Lists of differentially expressed genes were further annotated with pathway information from the KEGG database [91].

The association of Gene Ontology (GO) terms and KEGG metabolic pathways to genes in the list of differentially expressed genes (DEGs) resulting from comparisons of infected and uninfected mouse was assessed with functions from the R package *GOstats* [92]. For the applied conditional hypergeometric test for overrepresentation of GO terms in each of the three ontologies (molecular function, biological process and cellular component) and annotated KEGG pathways we used a p-value cut-off of 0.001. Mouse genes were classified as being differentially expressed if and only if |log_2_FC|> = 1.5 and (multiple testing corrected) p-value< = 0.05 hold. The used GO annotations were obtained from the Bioconductor *Mus musculus* annotation package, whereas KEGG pathway annotations were directly retrieved from KEGG using KEGG's REST API. Results derived from overrepresentation analyses are given in **S2** Table.

To assess platform dynamic range and the accuracy of fold-change response, we used ERCC RNA Spike-In Controls. Spike-in control sequences were added to mouse reference genome/annotation prior to read alignment and read counts for spike-in controls were determined along with normal gene counts with program *htseq-count*. Further data analyses and generation of dose- and fold-change-response plots were performed as described by the manufacturer (ERCC RNA- Spike-In Control Mixes User Guide: https://tools.thermofisher.com/content/sfs/manuals/cms_086340.pdf).

### Data access

FASTQ files, files containing gene counts determined by *htseq-count* of all libraries used in this study and lists of identified differentially expressed genes from the different comparisons are deposited in NCBI’s Gene Expression Omnibus (GEO) with the accession GSE98802.

### Quantitative real-time PCR (qRT-PCR)

qRT-PCR was employed to assess bacterial expression patterns by 3-step cycling using the SensiFast SYBR No-ROX One-Step kit (Bioline). DNA-depleted RNA was adjusted to a final concentration of 25 ng/μl and reverse-transcribed into cDNA at 45°C for 20 min as described by the manufacturer. Reverse transcription and subsequent qRT-PCRs were performed in the Rotor-Gene Q real-time PCR cycler (QIAGEN). A 3-step-cycling program (denaturation: 10 sec, 95°C, annealing: 52–62°C, 20 sec; polymerization: 72°C, 10–30 sec, < 50 cycles) with subsequent melt-profile analysis (58–99°C) to monitor product specificity was applied. The acquired data were processed with the Gene-Rotor Q Series software as described [22].

For bacterial expression analysis, *sopB* and *if-3* genes were used for normalization. Relative target gene expression compared to a reference gene was calculated according to [93]. Primers are listed in **S3** Table. KEGG accession for qRT-PCR tested transcripts: *sopB* (pYV0031), *wrbA* (YPK_2363), *hdeB* (YPK_1140), *cnfY* (YPK_2615), *csrA* (YPK_3372), *rovA* (YPK_1876), *crp* (YPK_0248), *yscF* (pYV0082), *yopJ* (pYV_0098), *yopE* (pYV0025), *yopH* (pYV0094), *yopD* (pYV0054), *ypkA* (pYV0001), *frdA* (YPK_3813), *rfaH* (YPK_3937), *arcA* (YPK_3606), *napA* (YPK_1387), *if-3* (YPK_1821)

### Statistical analysis

Graph Pad Prism 6.0g was used for statistical analysis of the data. For the statistical analysis of two groups the Mann-Whitney U test was executed. Column based data containing more than 2 groups was compared using One-way ANOVA. To compare two groups at different time points multiple t-tests were performed. For all multiple testing scenarios, the reported p-values were adjusted accordingly. Correlations were performed using the Spearman correlation method. Survival data was statistically analyzed with the Mantel-Cox log-rank test.

## Supporting information

S1 Fig Histopathology score of YPIII and YP147(Δ*cnfY*) infected cecal tissue. The inflammation score of H&E stained sections of the cecal lamina propria (**A**) and the cecal lymphoid tissue (**B**) of uninfected and infected BALB/c mice at 3 or 42 dpi with about 10^5−^10^6^ CFUs of YPIII or YP147(Δ*cnfY*)*/*g tissue. The data show the median scores of 5 mice and were statistically analyzed with multiple t-tests using Holm-Šídák correction: * p < 0.01.
(TIF)

S2 Fig Analysis of growth, virulence and persistence of mRuby2-expressing *Y. pseudotuberculosis*. **(A)** YPIII or YP147(Δ*cnfY*) mRuby2 expressing isogenic strains were grown at 25°C and 37°C in LB medium. At indicated time points, optical density at 600 nm was determined. The data show the mean +/- SEM of three independent experiments performed in duplicates. (**B-D**) The BALB/c mice were orally infected with 2x10^8^ CFU of YPIII or YP147(Δ*cnfY*) and their isogenic mRuby2-expressing strains and their health status was monitored over 14 days. The presented data represent two independent experiments with n = 8–10 per group. (**B**) Survival of BALB/c mice. Data were analyzed with the log-rank (Mantel-Cox) test, ns: not significant; ***: p < 0.001. (**C**) Weight loss of infected mice. Mice that lost more than 20% of their initial body weight were sacrificed and recorded as dead. The data represent the mean +/- SD and were analyzed with multiple t-tests using Holm-Šídák correction*;* *: p < 0.05. (**D**) Number of bacteria in the cecum at day 3 and 5 post infection. Statistical analysis was performed using the Kruskal-Wallis test and Dunn's correction; ns, not significant. (**E-G**) The BALB/c mice were orally infected with 1x10^6^ CFU of YPIII/YP147(Δ*cnfY*) or their isogenic Ruby2 expressing strains and their health status and bacterial loads in the feces were monitored over 42 days. (**E**) Relative body weight compared to the initial weight. The data show the mean YPIII n = 20; YP147(Δ*cnfY*) n = 10; YPIII *mRuby2* n = 40, YP147(Δ*cnfY*) *mRuby2* n = 20. Data were analyzed with multiple t-tests using Holm-Šídák correction; no significant differences were observed. *Yersinia* loads in the feces of infected mice with YPIII, YPIII (mRuby2) (**F**) or YP147(Δ*cnfY*), YP147(Δ*cnfY*) (mRuby2) (**G**) were determined at indicated time points. The bar illustrates the geometric mean. The data represent two independent experiments analyzed with the Mann-Whitney U test. The results were not significant; YPIII n = 40; YP147(Δ*cnfY*) n = 10; YPIII *mRuby2* n = 40, YP147(Δ*cnfY*) *mRuby2* n = 20.
(TIF)

S3 Fig Fecal microbiota in wildtype- and Δ*cnfY* mutant-infected mice. At indicated time points prior (-1) and post infection, feces was sampled from individual mice and tested for *Y*. *pseudotuberculosis*. The microbiota composition was analyzed by 16S rRNA gene sequencing and permutational multivariate analysis of variance (ADONIS) was used to calculate the variance explained by individual factors. Principal coordinates analysis (PCoA) was used to visualize β diversity globally and the bar plot displays the contribution of variables to the observed variance over one time point (**A**: prior to infection; **B**: 3 dpi, **C**: 9 dpi, **D**: 21 dpi, and **E**: 42 dpi). (**F**) Bar plot showing individual contribution of variables, including different strains (genotype), to the observed variance (calculated R^2^) at indicated time points. A significant effect was attributed when P-value is < 0.05 and R^2^ is > 0.01 (equivalent to 1% of explained variance); P-value: *** <0.001 ** <0.01, * <0.05.
(TIF)

S4 Fig Tissue RNA-Seq work flow and RNA isolation of *Yersinia*-infected ceca. (**A**) Host transcriptome assessment workflow of ceca from *Y*. *pseudotuberculosis* YPIII and YPIII Δ*cnfY*-infected mice or equally aged uninfected mice. Total RNA was isolated from the ceca of mice, processed for preparation of strand-specific barcoded cDNA libraries and sequenced. cDNA reads were separated *in silico* by mapping to the mm10 genome. (**B**) Representative Bioanalyzer profile of total RNA pools extracted from cecal tissue from uninfected, YPIII- and YP147(Δ*cnfY*)-infected mice during acute and persistent infection stage. The RIN indicates the quality of the total RNA pools. (**C**) Analysis of the bacterial load of the ceca at day 5 and 42 post infection with the wildtype and isogenic Δ*cnfY* mutant strain. BALB/c mice were intra-gastrically challenged with YPIII or YP147(Δ*cnfY*) for RNA-seq analysis (10^6^−10^7^ CFUs/g tissue). Mice were sacrificed after 5 days (acute infection) and 42 days (persistent infection) post infection and the number of bacteria in the cecal tissue was determined by plating. The median of the data is shown. Statistical analysis of the data was performed with One-way ANOVA employing Holm-Šídák’s correction. No significant differences were found.
(TIF)

S5 Fig RNA-seq platform performance. (**A-B**) ERCC RNA Spike-In Control mix analysis to determine the platform performance. (**A**) Platform dynamic range and lower limit of detection (LLD) (dose response). Either ERCC ExFold RNA Spike-In Mix 1 or Mix 2 was added to RNA pools obtained from infected and uninfected cecal lymphoid tissue. Column 1, 2 and 3 represents replicates 1, 2 and 3. (**B**) Fold change plots are the result of two libraries of independent replicates. Assessment of platform fold-change responses shows linearity between read intensity and RNA input and demonstrates accuracy. ERCC ExFold RNA Spike-In Mix 1 or Mix 2 was added to mouse RNA pools, which were then converted into cDNA libraries and sequenced. The observed fold-change ratios between Mix 1 and Mix 2 should match with the expected ratios, which can be determined by linear regression. Controls with an RPKM ≤ 1 (open circles) were removed in either sample and the linear fit illustrates highly accurate fold-change estimates (filled circles; R^2^ = 0.956–0.982).
(TIF)

S6 Fig RNA-seq data reproducibility between replicates. RPKM normalized read counts for all detected mouse genes of uninfected, YPIII- and YP147(Δ*cnfY*)-infected mice during acute and persistent infection stage are plotted for all the biological replicates. The Pearson correlation coefficient (*r*) is given for each replicate.
(TIF)

S7 Fig Host transcriptional changes upon a wildtype and Δ*cnfY* mutant infection. Volcano plots obtained from *DESeq2* analysis of uninfected and infected cecal RNA pools obtained from acute (**A**) and persistently (**B**) infected mice.
(TIF)

S8 Fig Expression pattern of persistence-relevant *Yersinia* genes. Relative changes in transcript abundance of selected fitness-relevant *Yersinia* genes were determined from RNA isolated from (**A**) YPIII- or YP147(Δ*cnfY*)-infected ceca 5 and 42 dpi, or (**B**) from bacteria grown *in vitro* at 25°C and 37°C. qRT-PCR was performed in four technical replicates. Bacterial transcript abundance of *sopB* and *if-3* were used for normalization. The data show the mean +/- SEM of at least three independent experiments performed in two (persistent phase) or four (acute phase) technical replicates and were analyzed by multiple t-tests employing Holm-Šídák’s correction, P-value: *<0.05.
(TIF)

S1 Table Mapping statistics of RNA-seq libraries. (DOCX)

S2 Table Host processes regulated during acute infection with *Y. pseudotuberculosis* (5 dpi). (DOCX)

S3 Table Strains, plasmids and primers. (DOCX)

S1 Dataset Global gene expression changes within BALB/c mice (DESeq2 analysis cecum uninfected vs. YPIII infected at 5 dpi). (XLS)

S2 Dataset Global gene expression changes within BALB/c mice (DESeq2 analysis cecum uninfected vs. YP147 (Δ*cnfY*) at 5 dpi). (XLS)

S3 Dataset Global gene expression changes within BALB/c mice (DESeq2 analysis cecum YPIII vs YP147 (Δ*cnfY*) at 5 dpi). (XLS)

S4 Dataset Global gene expression changes within BALB/c mice (DESeq2 analysis cecum uninfected vs. YPIII infected at 42 dpi). (XLS)

S5 Dataset Global gene expression changes within BALB/c mice (DESeq2 analysis cecum uninfected vs. YP147 (Δ*cnfY*) at 42 dpi). (XLS)

S6 Dataset Global gene expression changes within BALB/c mice (DESeq2 analysis cecum YP147 (Δ*cnfY*) vs. YPIII at 42 dpi). (XLS)
