# Supplementary figures and images for: Loss of CNFY toxin-induced inflammation drives Yersinia pseudotuberculosis into persistency
Source: PLoS Pathog. 2018 Feb 1;14(2):e1006858. doi: 10.1371/journal.ppat.1006858 (PMC5811047; doi:10.1371/journal.ppat.1006858)

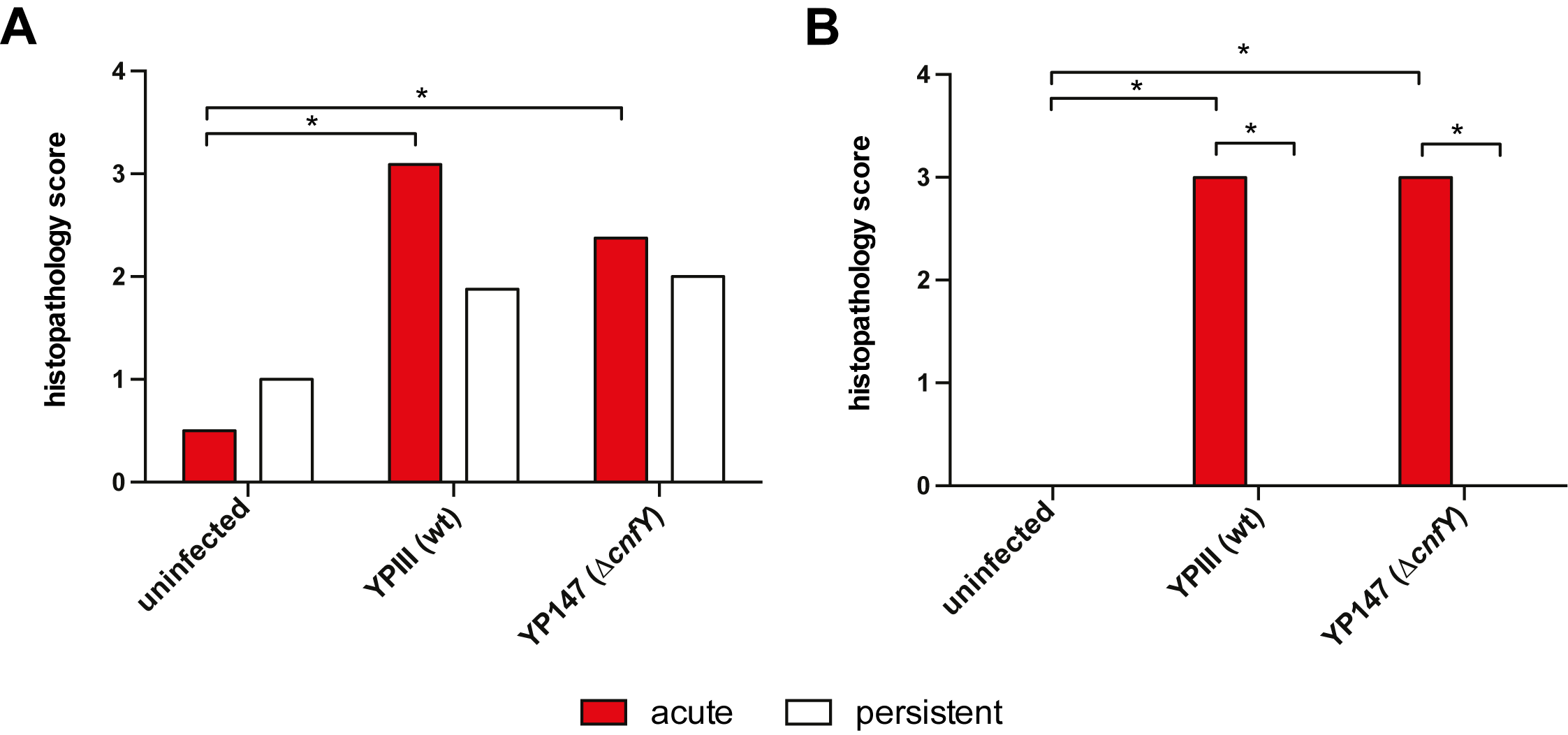

Supplement: S1 Fig — The inflammation score of H&E stained sections of the cecal lamina propria (A) and the cecal lymphoid tissue (B) of uninfected and infected BALB/c mice at 3 or 42 dpi with about 105−106 CFUs of YPIII or YP147(ΔcnfY)/g tissue. The data show the median scores of 5 mice and were statistically analyzed with multiple t-tests using Holm-Šídák correction: * p < 0.01. (TIF) [file ppat.1006858.s001.tif]

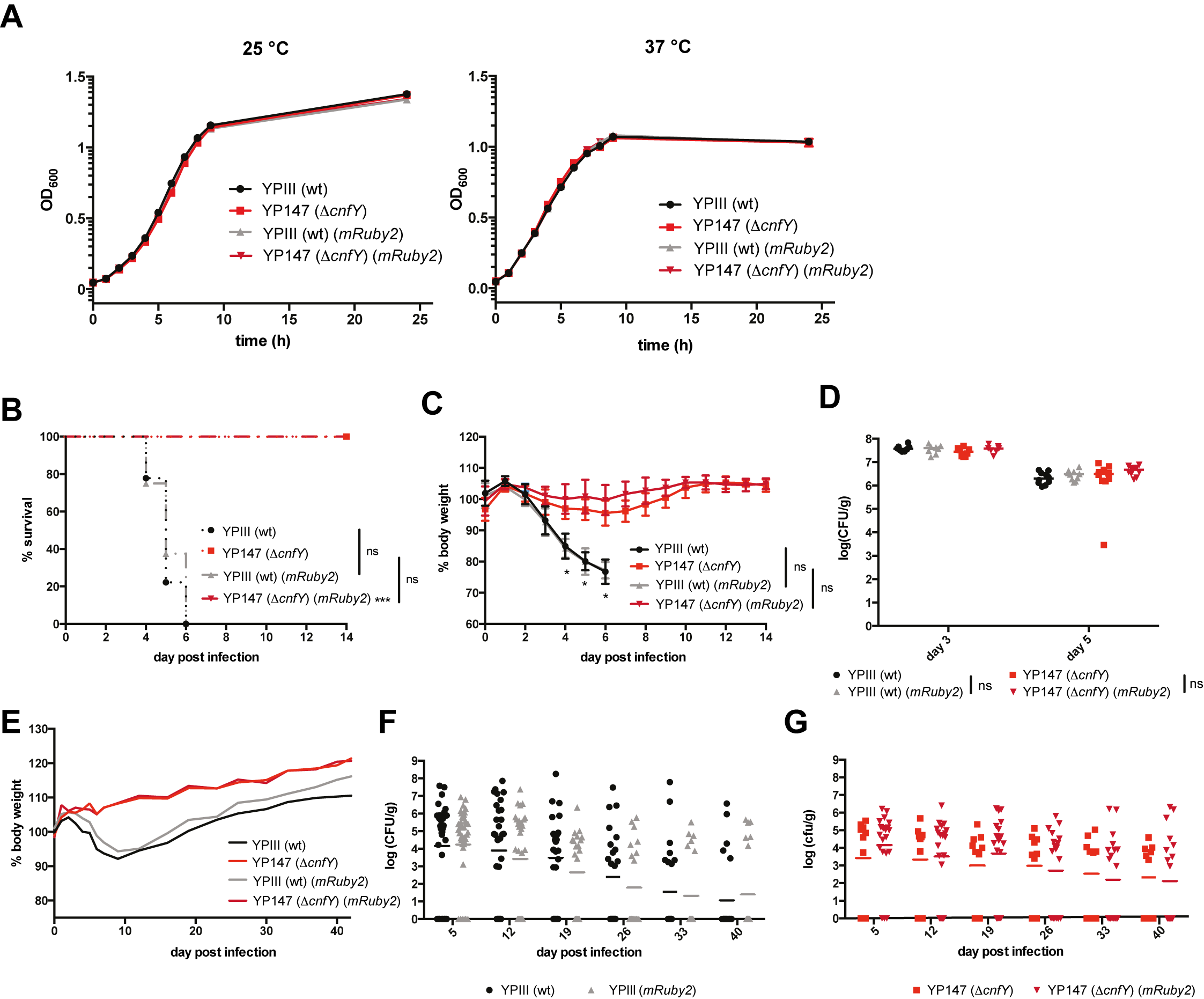

Supplement: S2 Fig — (A) YPIII or YP147(ΔcnfY) mRuby2 expressing isogenic strains were grown at 25°C and 37°C in LB medium. At indicated time points, optical density at 600 nm was determined. The data show the mean +/- SEM of three independent experiments performed in duplicates. (B-D) The BALB/c mice were orally infected with 2x10^8^ CFU of YPIII or YP147(ΔcnfY) and their isogenic mRuby2-expressing strains and their health status was monitored over 14 days. The presented data represent two independent experiments with n = 8–10 per group. (B) Survival of BALB/c mice. Data were analyzed with the log-rank (Mantel-Cox) test, ns: not significant; ***: p < 0.001. (C) Weight loss of infected mice. Mice that lost more than 20% of their initial body weight were sacrificed and recorded as dead. The data represent the mean +/- SD and were analyzed with multiple t-tests using Holm-Šídák correction; *: p < 0.05. (D) Number of bacteria in the cecum at day 3 and 5 post infection. Statistical analysis was performed using the Kruskal-Wallis test and Dunn's correction; ns, not significant. (E-G) The BALB/c mice were orally infected with 1x10^6^ CFU of YPIII/YP147(ΔcnfY) or their isogenic Ruby2 expressing strains and their health status and bacterial loads in the feces were monitored over 42 days. (E) Relative body weight compared to the initial weight. The data show the mean YPIII n = 20; YP147(ΔcnfY) n = 10; YPIII mRuby2 n = 40, YP147(ΔcnfY) mRuby2 n = 20. Data were analyzed with multiple t-tests using Holm-Šídák correction; no significant differences were observed. Yersinia loads in the feces of infected mice with YPIII, YPIII (mRuby2) (F) or YP147(ΔcnfY), YP147(ΔcnfY) (mRuby2) (G) were determined at indicated time points. The bar illustrates the geometric mean. The data represent two independent experiments analyzed with the Mann-Whitney U test. The results were not significant; YPIII n = 40; YP147(ΔcnfY) n = 10; YPIII mRuby2 n = 40, YP147(ΔcnfY) mRuby2 n = 20. (TIF) [file ppat.1006858.s002.tif]

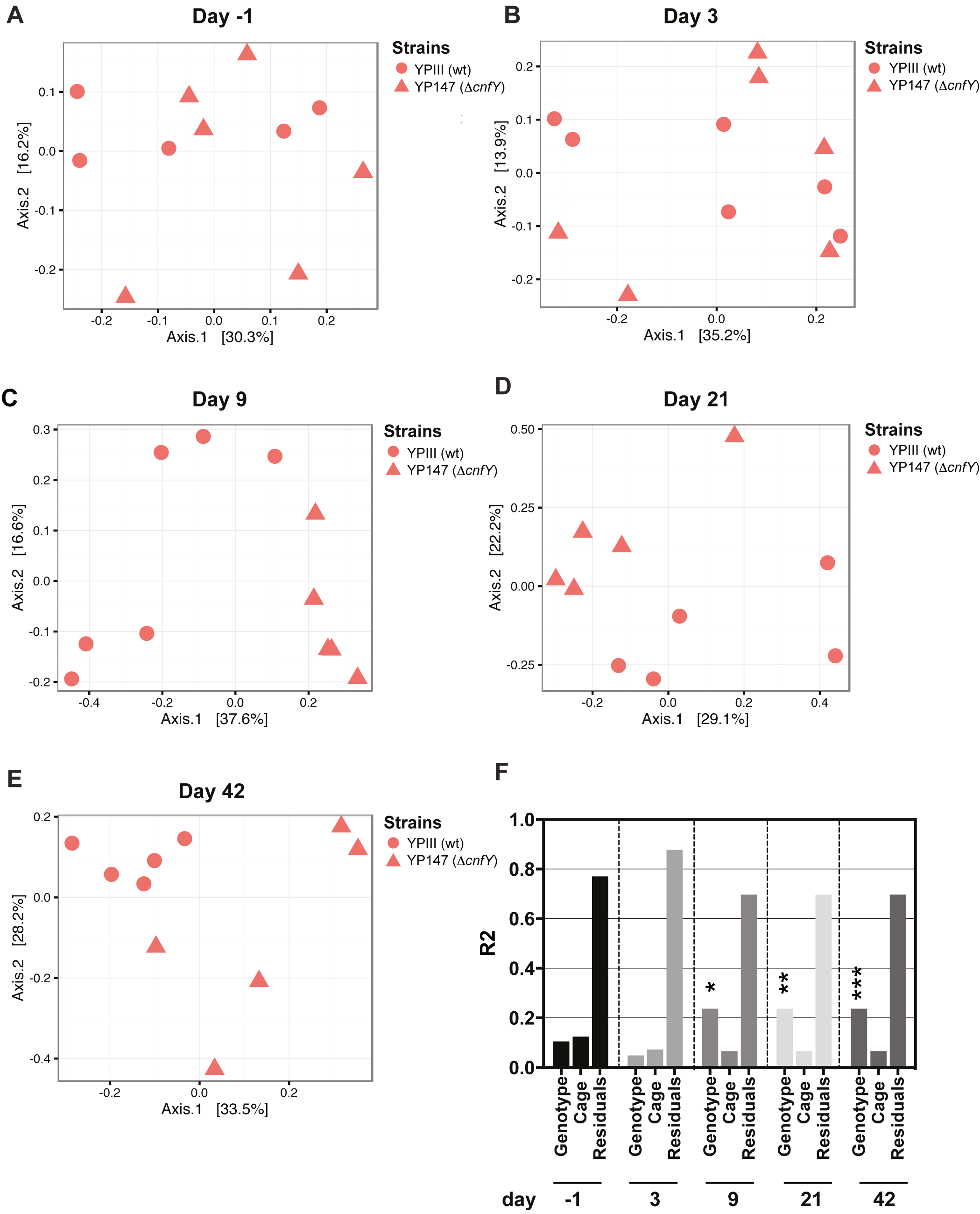

Supplement: S3 Fig — At indicated time points prior (-1) and post infection, feces was sampled from individual mice and tested for Y. pseudotuberculosis. The microbiota composition was analyzed by 16S rRNA gene sequencing and permutational multivariate analysis of variance (ADONIS) was used to calculate the variance explained by individual factors. Principal coordinates analysis (PCoA) was used to visualize β diversity globally and the bar plot displays the contribution of variables to the observed variance over one time point (A: prior to infection; B: 3 dpi, C: 9 dpi, D: 21 dpi, and E: 42 dpi). (F) Bar plot showing individual contribution of variables, including different strains (genotype), to the observed variance (calculated R2) at indicated time points. A significant effect was attributed when P-value is < 0.05 and R2 is > 0.01 (equivalent to 1% of explained variance); P-value: *** <0.001 ** <0.01, * <0.05. (TIF) [file ppat.1006858.s003.tif]

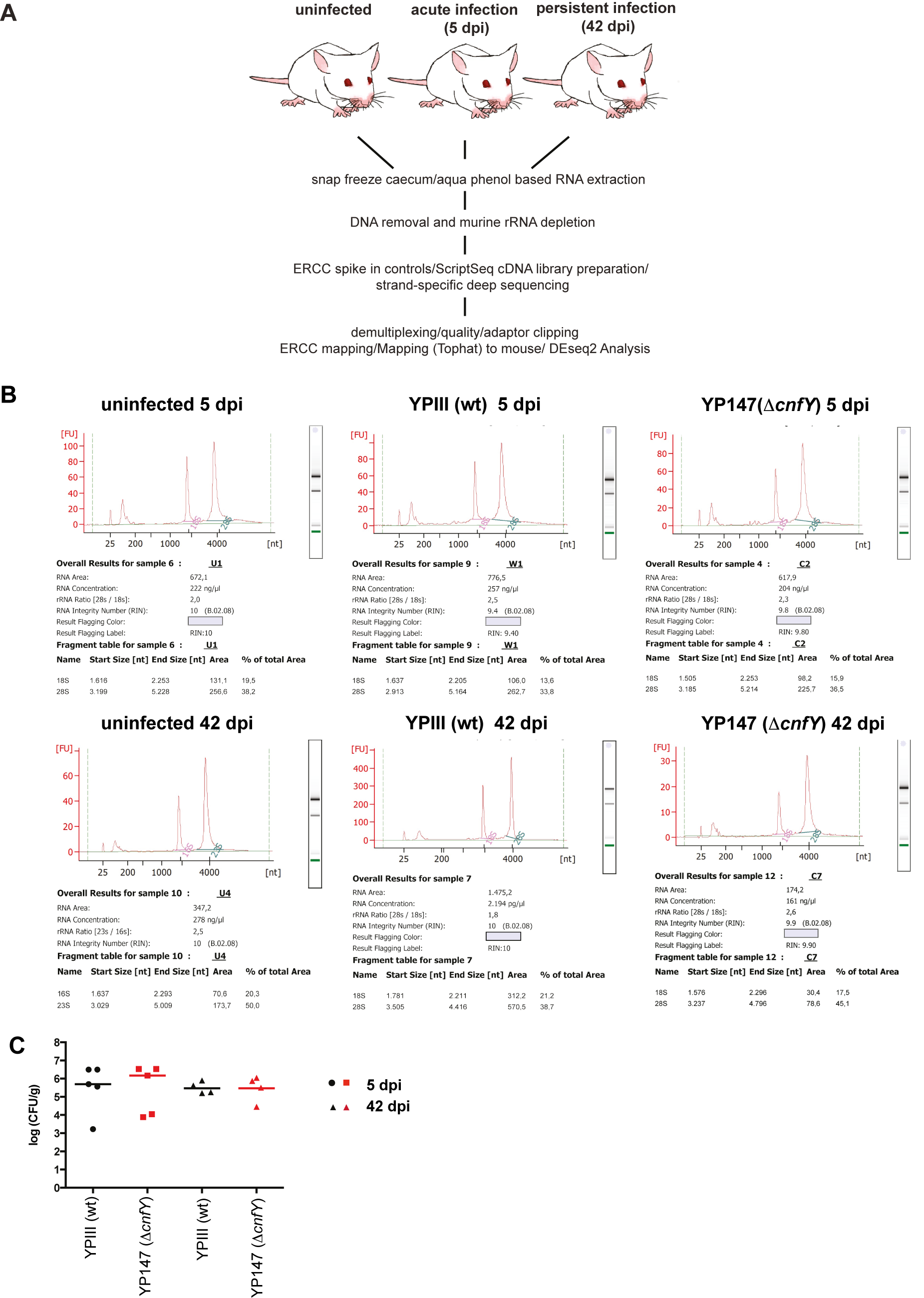

Supplement: S4 Fig — (A) Host transcriptome assessment workflow of ceca from Y. pseudotuberculosis YPIII and YPIII ΔcnfY-infected mice or equally aged uninfected mice. Total RNA was isolated from the ceca of mice, processed for preparation of strand-specific barcoded cDNA libraries and sequenced. cDNA reads were separated in silico by mapping to the mm10 genome. (B) Representative Bioanalyzer profile of total RNA pools extracted from cecal tissue from uninfected, YPIII- and YP147(ΔcnfY)-infected mice during acute and persistent infection stage. The RIN indicates the quality of the total RNA pools. (C) Analysis of the bacterial load of the ceca at day 5 and 42 post infection with the wildtype and isogenic ΔcnfY mutant strain. BALB/c mice were intra-gastrically challenged with YPIII or YP147(ΔcnfY) for RNA-seq analysis (106−107 CFUs/g tissue). Mice were sacrificed after 5 days (acute infection) and 42 days (persistent infection) post infection and the number of bacteria in the cecal tissue was determined by plating. The median of the data is shown. Statistical analysis of the data was performed with One-way ANOVA employing Holm-Šídák’s correction. No significant differences were found. (TIF) [file ppat.1006858.s004.tif]

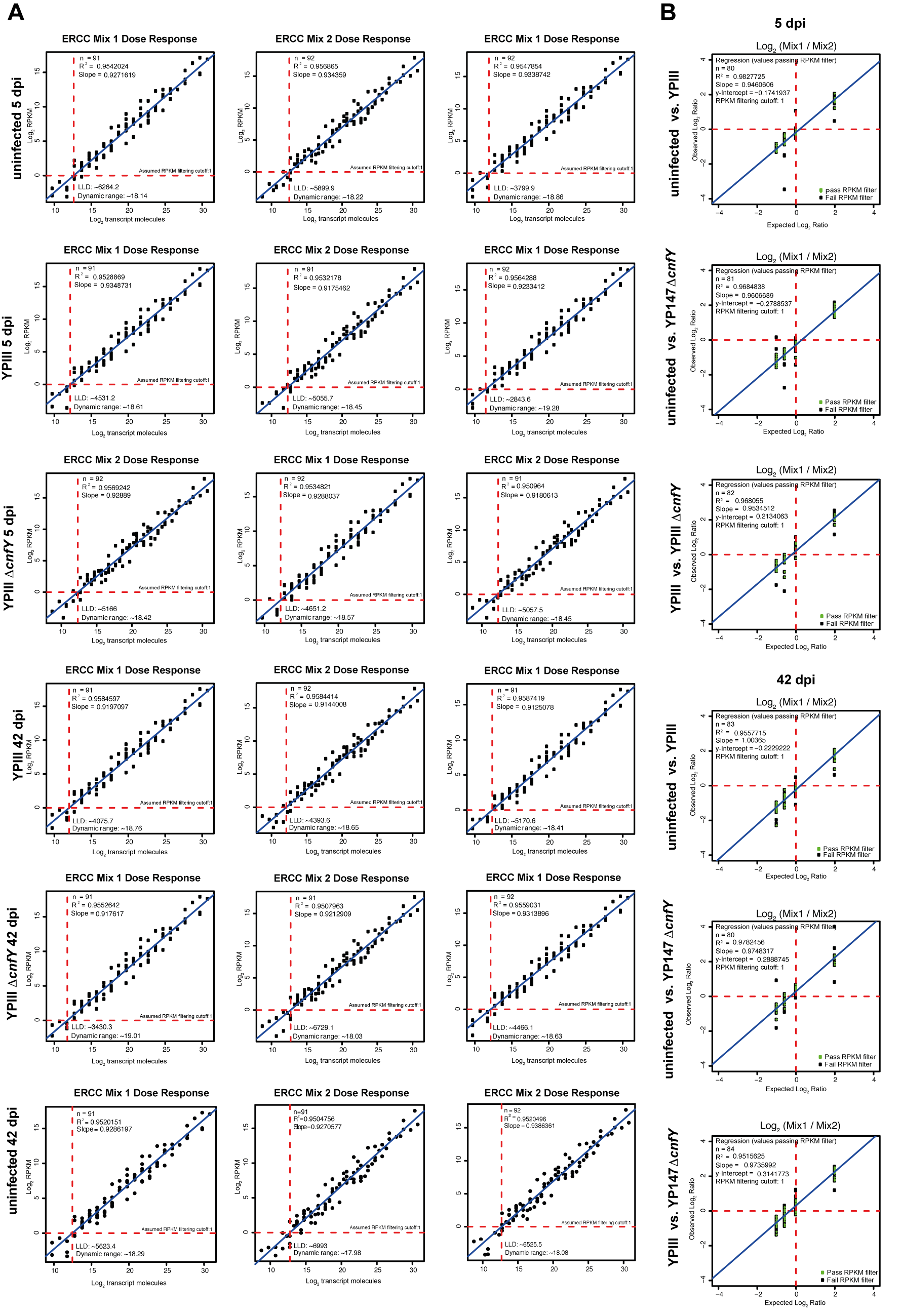

Supplement: S5 Fig — (A-B) ERCC RNA Spike-In Control mix analysis to determine the platform performance. (A) Platform dynamic range and lower limit of detection (LLD) (dose response). Either ERCC ExFold RNA Spike-In Mix 1 or Mix 2 was added to RNA pools obtained from infected and uninfected cecal lymphoid tissue. Column 1, 2 and 3 represents replicates 1, 2 and 3. (B) Fold change plots are the result of two libraries of independent replicates. Assessment of platform fold-change responses shows linearity between read intensity and RNA input and demonstrates accuracy. ERCC ExFold RNA Spike-In Mix 1 or Mix 2 was added to mouse RNA pools, which were then converted into cDNA libraries and sequenced. The observed fold-change ratios between Mix 1 and Mix 2 should match with the expected ratios, which can be determined by linear regression. Controls with an RPKM ≤ 1 (open circles) were removed in either sample and the linear fit illustrates highly accurate fold-change estimates (filled circles; R2 = 0.956–0.982). (TIF) [file ppat.1006858.s005.tif]

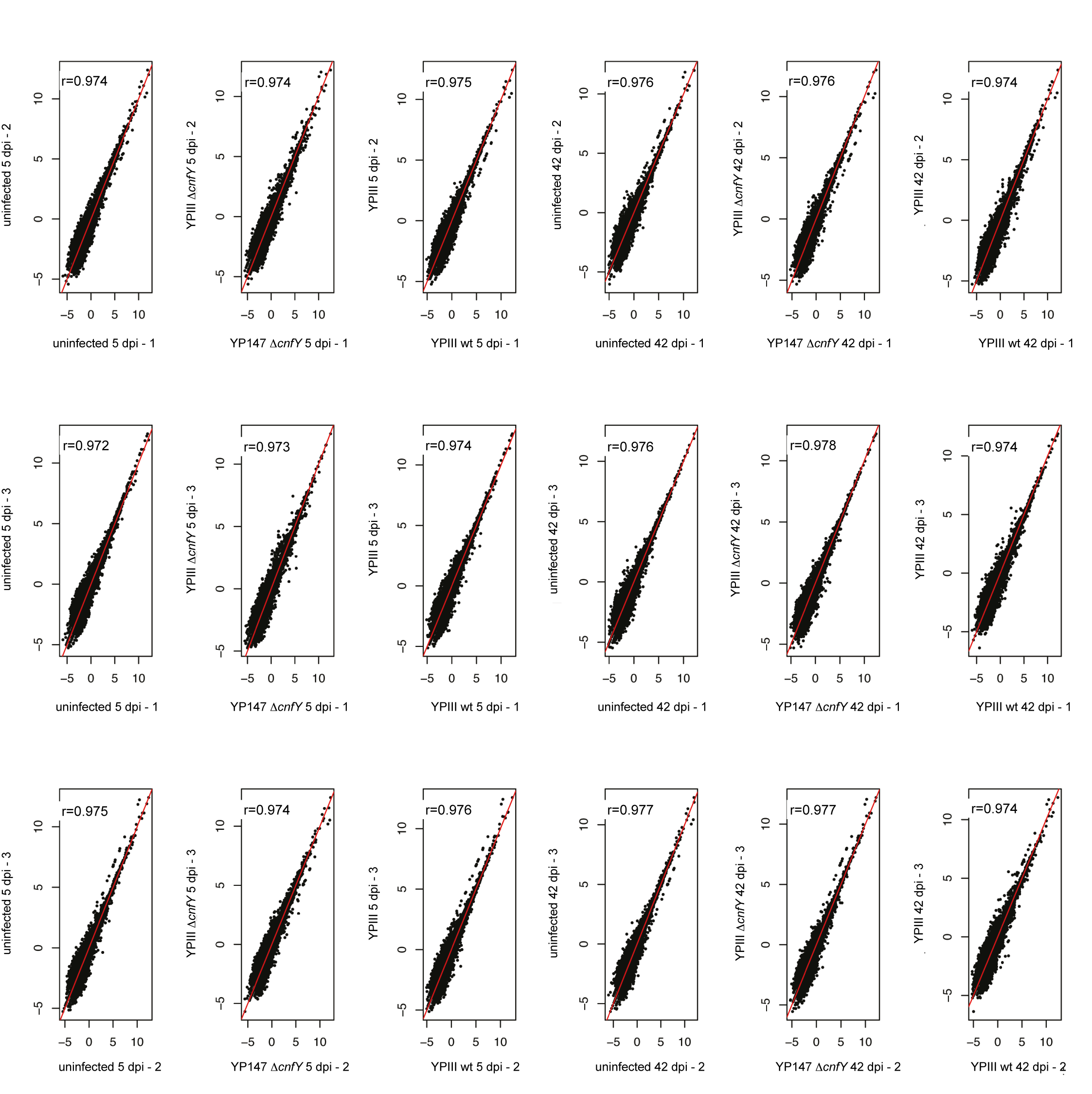

Supplement: S6 Fig — RPKM normalized read counts for all detected mouse genes of uninfected, YPIII- and YP147(ΔcnfY)-infected mice during acute and persistent infection stage are plotted for all the biological replicates. The Pearson correlation coefficient (r) is given for each replicate. (TIF) [file ppat.1006858.s006.tif]

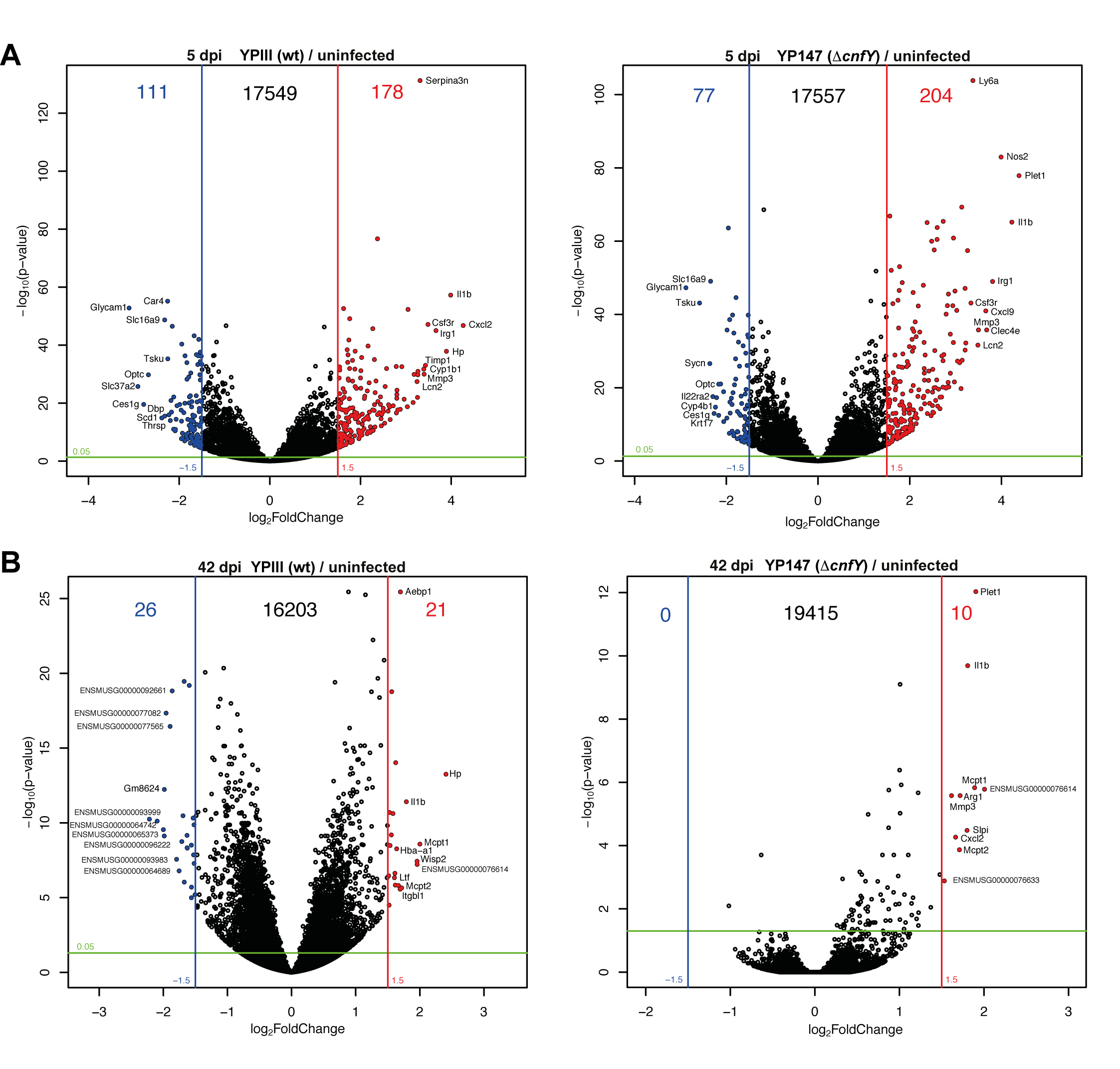

Supplement: S7 Fig — Volcano plots obtained from DESeq2 analysis of uninfected and infected cecal RNA pools obtained from acute (A) and persistently (B) infected mice. (TIF) [file ppat.1006858.s007.tif]

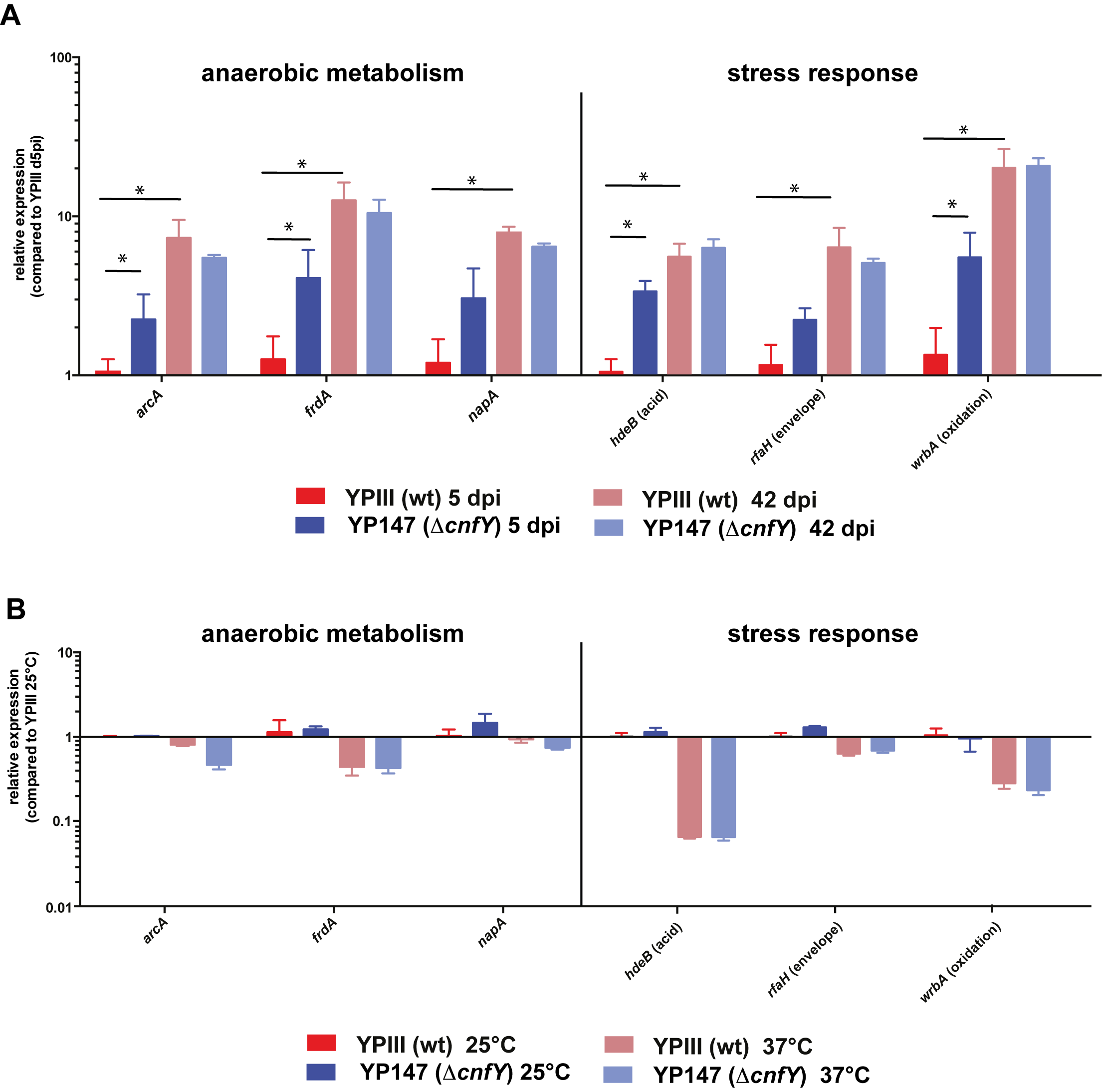

Supplement: S8 Fig — Relative changes in transcript abundance of selected fitness-relevant Yersinia genes were determined from RNA isolated from (A) YPIII- or YP147(ΔcnfY)-infected ceca 5 and 42 dpi, or (B) from bacteria grown in vitro at 25°C and 37°C. qRT-PCR was performed in four technical replicates. Bacterial transcript abundance of sopB and if-3 were used for normalization. The data show the mean +/- SEM of at least three independent experiments performed in two (persistent phase) or four (acute phase) technical replicates and were analyzed by multiple t-tests employing Holm-Šídák’s correction, P-value: *<0.05. (TIF) [file ppat.1006858.s008.tif]
