# Supplementary material for: Loss of CNFY toxin-induced inflammation drives Yersinia pseudotuberculosis into persistency
Source: PLoS Pathog. 2018 Feb 1;14(2):e1006858. doi: 10.1371/journal.ppat.1006858 (PMC5811047; doi:10.1371/journal.ppat.1006858)
Supplement: S1 Table — (DOCX) [file ppat.1006858.s009.docx]

**Table S1:** Mapping statistics of RNA-seq libraries

| Library Sample* | Total number of read counts^1^ | Number of reads mapped to  mm10 genome^2^ | Mapped to  mm10 genes | Mapped to ERCC |
| --- | --- | --- | --- | --- |
| 5 dpi uninfected 1 | 22,047,027 | 14,669,574 | 11,138,040 | 5,760,438 |
| 5 dpi uninfected 2 | 21,873,833 | 14,223,693 | 10,367,252 | 5,828,754 |
| 5 dpi uninfected 3 | 22,107,361 | 12,210,972 | 8,657,003 | 8,133,512 |
| 5 dpi YPIII 1 | 18,014,829 | 10,540,510 | 7,211,779 | 6,049,272 |
| 5 dpi YPIII 2 | 23,817,131 | 15,347,411 | 10,181,473 | 6,423,910 |
| 5 dpi YPIII 3 | 26,875,039 | 13,632,231 | 8,654,807 | 10,900,993 |
| 5 dpi YPIII Δ*cnfY* 1 | 26,009,339 | 16,411,709 | 11,073,180 | 7,373,373 |
| 5 dpi YPIII Δ*cnfY* 2 | 19,350,404 | 11,466,580 | 8,342,268 | 6,199,475 |
| 5 dpi YPIII Δ*cnfY* 3 | 19,536,186 | 12,440,019 | 8,207,709 | 5,275,159 |
| 42 dpi uninfected 1 | 24,908,621 | 17,182,363 | 12,736,288 | 5,637,693 |
| 42 dpi uninfected 2 | 27,407,426 | 17,482,471 | 12,846,119 | 7,704,677 |
| 42 dpi uninfected 3 | 27,665,624 | 18,191,840 | 12,969,359 | 6,881,262 |
| 42 dpi YPIII 1 | 39,795,513 | 23,818,604 | 14,934,833 | 12,658,943 |
| 42 dpi YPIII 2 | 38,071,123 | 24,552,069 | 16,591,558 | 10,647,313 |
| 42 dpi YPIII 3 | 38,140,082 | 24,762,343 | 16,507,236 | 9,982,955 |
| 42 dpi YPIII Δ*cnfY* 1 | 25,239,634 | 13,956,701 | 10,008,228 | 9,023,717 |
| 42 dpi YPIII Δ*cnfY* 2 | 24,297,789 | 16,606,374 | 11,899,990 | 5,397,356 |
| 42 dpi YPIII Δ*cnfY* 3 | 27,823,321 | 16,275,006 | 11,698,916 | 9,051,417 |

Read length: 51 nt, genomic mapping rate: 50-68%, mapping rate with ERCC 91-93%

*Data deposit: Gene expression omnibus (GEO) accession GSE98802.

^1^Sequencing technology: Illumina HiSeq2000

^2^cDNA reads were mapped to the *Mus musculus* mm10 genome using TopHat2.
