# Supplementary material for: Loss of CNFY toxin-induced inflammation drives Yersinia pseudotuberculosis into persistency
Source: PLoS Pathog. 2018 Feb 1;14(2):e1006858. doi: 10.1371/journal.ppat.1006858 (PMC5811047; doi:10.1371/journal.ppat.1006858)
Supplement: S2 Table — (DOCX) [file ppat.1006858.s010.docx]

**Table S2:** Host processes regulated during acute infection with *Y. pseudotuber­culosis* (5 dpi)

| **Regulation** | **Term/Pathway** | **Percentage OddsRatio** | ***P* value** |
| --- | --- | --- | --- |

**YPIII/uninfected**

**GO enrichment analysis**

| 1 | CXCR chemokine receptor binding | 76,5 | 3.2E-09 |
| --- | --- | --- | --- |
| 2 | leukocyte migration involved in inflammatory response | 55.3 | 2.2E-07 |
| 3 | positive regulation of monocyte chemotaxis | 49.1 | 3.4E-07 |
| 4 | fever generation | 39.2 | 1.1E-05 |
| 5 | NLP3 inflammasome complex assembly | 37.6 | 1.7E-04 |
| 6 | retinoic acid biosynthesis process | 32,9 | 2.3E-05 |
| 7 | chemokine binding | 32.2 | 2.0E-05 |
| 8 | chemokine activity | 31.2 | 1.4E-11 |
| 9 | chemokine-mediated signaling pathway | 28.2 | 3.8E-14 |
| 10 | regulation of monocyte chemotactic protein-1 production | 26.3 | 3.8E-04 |
| 11 | interleukin-1 receptor binding | 25.3 | 4.4E-05 |
| 12 | acute phase response | 24.6 | 5.8E-07 |
| 13 | CC chemokine receptor activity | 24.1 | 4.8E-04 |
|  |  |  |  |

**KEGG pathway enrichment analysis**

| 1 | IL-17 signaling pathway - Mus musculus (mouse) | 10.5 | 1.0E-09 |
| --- | --- | --- | --- |
| 2 | Osteoclast differentiation | 9.0 | 1.7E-10 |
| 3 | *Salmonella* infection | 8.2 | 1.6E-06 |
| 4 | Malaria | 7.6 | 2.7E-04 |
| 5 | Legionellosis | 7.5 | 9.2E-05 |
| 6 | Linoleic acid metabolism | 7.4 | 3.1E-04 |
| 7  8  9 | *Staphylococcus aureus* infection  Pertussis  Hematopoietic cell lineage | 7.1  6.6  6.6 | 3.8E-04  7.1E-05  9.7E-06 |
| 10  11  12  11  12 | Arachidonic acid metabolism  Chemical carcinogenesis  Cytokine-cytokine receptor interaction  Rheumatoid arthritis  Toll-like receptor signaling pathway | 6.2  5.9  5.9  5.8  5.5 | 3.8E-05  5.9E-05  1.3E-10  1.5E-04  8.9E-05 |
| 13  14 | Amoebiasis  TNF signaling pathway | 5.1  5.1 | 1.5E-04  1.6E-04 |
| 15  16 | Chemokine signaling pathways  NOD-like receptor signaling pathway | 4.9  4.4 | 2.6E-06  6.2E-05 |
|  |  |  |  |

**YPIII Δ*cnfY* / uninfected**

**GO enrichment analysis**

| 1 | CXCR chemokine receptor binding | 80.0 | 2.5E-09 |
| --- | --- | --- | --- |
| 2 | chemokine metabolic process | 58.7 | 1.7E-07 |
| 3 | cellular response to interferon-γ | 55.7 | 8.3E-15 |
| 4 | acute phase response | 54.4 | 1.6E-13 |
| 5 | adhesion to symbiont to host | 47.0 | 3.8E-07 |
| 6 | defense response to protozoan | 43.0 | 1.4E-11 |
| 7 | leukocyte migration involved in inflammatory response | 41.6 | 8.6E-06 |
| 8 | regulation of cell killing | 40.7 | 1.3E-04 |
| 9 | NLRP3 inflammasome complex assembly | 39.9 | 1.4E-04 |
| 10 | positive regulation of neutrophil chemotaxis | 36.8 | 6.2E-09 |
| 11 | chemokine binding | 33.7 | 1.7E-05 |
| 12 | chemokine activity | 32.7 | 9.1E-12 |
| 13 | chemokine-mediated signaling pathway | 30.0 | 1.8E-14 |
| 14 | positive regulation of interleukin-1β secretion | 28.8 | 2.8E-05 |
| 15 | regulation of neutrophil migration | 27.2 | 3.7E-09 |
| 16 | interleukin-1 receptor binding | 26.4 | 3.7E-05 |

**KEGG pathway enrichment analysis**

| 1 | IL-17 signaling pathway - Mus musculus (mouse) | 13.1 | 3.9E-12 |
| --- | --- | --- | --- |
| 2 | *Salmonella* infection | 9.7 | 1.1E-07 |
| 3 | African trypanosomiasis | 9.1 | 4.1E-04 |
| 4 | TNF signaling pathway | 9.1 | 5.3E-09 |
| 5 | Rheumatoid arthritis | 8.0 | 2.0E-06 |
| 6 | NOD-like receptor signaling pathway | 8.0 | 9.8E-11 |
| 7 | Malaria | 8.0 | 2.2E-04 |
| 8 | Legionellosis | 7.8 | 7.1E-05 |
| 9  10 | Leishmaniasis  Hematopoietic cell lineage | 7.8  6.9 | 2.3E-05  6.8E-06 |
| 11 | Toll-like receptor signaling pathway | 6. 6 | 9.8E-06 |
| 12 | Inflammatory bowel disease (IBD) | 6.4 | 6.1E-04 |
| 13 | Osteoclast differentiation | 6.1 | 2.1E-06 |
| 14 | Amoebiasis | 6.1 | 1.8E-05 |
| 15 | Cytotosolic DNA-sensing pathway | 5.9 | 9.4E-04 |
| 16 | Cytokine-cytokine receptor interaction | 5.6 | 2.0E-09 |
| 17 | HIF-1 signaling pathway | 5.4 | 1.0E-04 |
| 18 | Chemokine signaling pathway | 5.1 | 1.5E-06 |
| 19 | Tuberculosis | 5.1 | 3.2E-06 |
| 20 | Chargas disease (American trypanosomiasis) | 4.9 | 4.3E-04 |
| 21 | Toxoplasmiosis | 4.6 | 6.7E-04 |
| 22 | Influenza | 4.5 | 4.4E-05 |
| 23 | Herpes simplex infection | 3.5 | 4.5E-04 |
|  |  |  |  |

GO: gene ontology. The top differentially infection-relevant pathways of the KEGG and the GO-enrichment from all three GOs biological processes, molecular function and cellular components were selected. Regulation was determined using RNA-seq technology. Results obtained from the *DESeq*2 tool were analyzed.
