# Supplementary material for: Loss of CNFY toxin-induced inflammation drives Yersinia pseudotuberculosis into persistency
Source: PLoS Pathog. 2018 Feb 1;14(2):e1006858. doi: 10.1371/journal.ppat.1006858 (PMC5811047; doi:10.1371/journal.ppat.1006858)
Supplement: S3 Table — (DOCX) [file ppat.1006858.s011.docx]

**Table S3:** Strains, plasmids and primers

| Strains | | Description | | Source/ Reference | |
| --- | --- | --- | --- | --- | --- |
| ***E. coli*** | | | | | |
| S17-1 λpir | | RP4-2 Tc::Mu-1 Km::Tn7 λpir, *pro82*, *recA1*, *endA1*, *thiE1*, *hsdR17*, *creC510* | | [83] | |
| ***Y. pseudotuberculosis*** | | | | | |
| YPIII | | pIB1, wildtype | | [84] | |
| YPIII Δ*cnfY* | | YP147, pIB1, *cnfY*::*kan* | | [11] | |
| YPIII (mRuby2) | | YP339, pIB1, P_L_*_tet_*_O-1_::*mRuby2* | | This study | |
| YPIII Δ*cnfY* (mRuby2) | | YP340, pIB1, *cnfY*::*kan*, P_L_*_tet_*_O-1_::*mRuby2* | | This study | |
|  | |  | |  | |
| Plasmids | | Description | | Source/ Reference | |
| pAKH3 | | R6K derivate, *sacB*, Amp^R^ | | [85] | |
| pFS43 | | pFS42 derivate, P_L_*_tet_*_O-1_::*mCherry*, Kan^R^ | | This study | |
| pWH9 | | pAKH3 derivate, *ypy*; YPK_3295 (-67 bp) to YPK_3294 (-1063 bp), Amp^R^ | | This study | |
| pWH14 | | pWH9 derivate, P_L_*_tet_*_O-1_::*mRuby2*, Amp^R^ | | This study | |
|  | |  | |  | |
| Oligonucleo-tides | | Sequence (5’ 🡪 3’) | | Description | |
| **PCR** | |  | |  | |
| II26 | | GCACGGTCGACATGGCCTCCTCCGAGAAC | |  | |
| II27 | | GCACTGCGGCCGCCTACAGGAACAGGTGGTGG | |  | |
| III981 | | TGAACGGCAGGTATATGTG | | Sequencing primer for pAKH3 | |
| III982 | | CACTTAACGGCTGACATGG | | Sequencing primer for pAKH3 | |
| VI392 | | GCGC**GAGCTC**TCATCTCGCACAACCAAGTTATA | | Sense primer upstream fragment for integration into the *Y. pseudo­tuberculosis* genome; creates *Sac*I site | |
| VI393 | | TTCATTTTCTAACTTCCGCT**CTCGAGGCGGCCGC**ATGTCATAAAAGGTAAATGGCATAATGG | | Sense primer downstream fragment for integration into the *Y. pseudo­tuberculosis* genome; creates *Not*I and *Xho*I sites | |
| VI394 | | CCATTTACCTTTTATGACAT**GCGGCCGCCTCGAG**AGCGGAAGTTAGAAAATGAAATTGA | | Antisense primer up­stream frag­ment for integration into the *Y. pseudotube­culosis* genome; creates *Xho*I and *Not*I sites | |
| VI395 | | GCGC**GAGCTC**TCTTTATTTTAACTTACTTGGCATAACTGG | | Antisense primer downstream frag­ment for integration into the *Y. pseudotuberculosis* genome; creates *Sac*I site | |
| VI504 | | GCCGAACCACAGTTATGGAGG | | Sequencing primer for chromosomal integration of P*_LtetO-1_*::*mRuby2* | |
| VI505 | | AGACACTGTAAGTCTCCTGTGATAGG | | Sequencing primer for chromosomal integration of P*_LtetO-1_*::*mRuby2* | |
| VI545 | | GCCC**CTCGAG**TCCCTATCAGTGATAGAGATTGACA | | Sense primer for P*_LtetO-1_*; creates *Xho*I site | |
| VI548 | | GCCC**GCGGCCGC**TCATTTATACAGTTCATCCATGCCG | | Antisense primer for *mRuby2*, creates *Not*I site | |
| VI556 | | TTTTTTTTCCTCCTTATTTTGTCGACGC | | Reverse primer for P*_LtetO-1_* | |
| VI557 | | AAAATAAGGAGGAAAAAAAAATGGTGAGCAAAGGTGAAGAGT | | Sense primer for *mRuby2* | |
|  | |  | |  | |
| ***Yersinia* qRT-PCR** | |  | |  | |
| III44 | | GAGACAACTCCACACCCAAAC | | Sense primer for *lcrF* (pYV0076) | |
| III45 | | GCAAAAGCAGTAATTCCTCAATAC | | Antisense primer for *lcrF* (pYV0076) | |
| III186 | | TGTAGTCGGGGACGTTATCG | | Sense primer for *gyrA* (YPK_2846) | |
| III187 | | CCCATCCACCAGCATATAGC | | Antisense primer for *gyrA* (YPK_2846) | |
| III393 | | CCGACGTAAAGCCGCGATAC | | Sense primer for *sopB* (pYV0031) | |
| III394 | | CCTCGTTCATAAGCACTCGTC | | Antisense primer for *sopB* (pYV0031) | |
| III788 | | GCTGAAACCGTTGGAACTGAC | | Sense primer for *rovA* (YPK_1876) | |
| III789 | | CTTCGCACGACGATCATTT | | Antisense primer for *rovA* (YPK_1876) | |
| IV549 | | CACCACACGAACTGGCCG | | Sense primer for *wrbA* (YPK_2363) | |
| IV550 | | CACGAGTCTGCGAGACATC | | Antisense primer for *wrbA* (YPK_2363) | |
| IV555 | | TTTCCACCGCGGCAACTACC | | Sense primer for *hdeB* (YPK_1140) | |
| IV556 | | CTTTCTGGGGCCTTCTTAC | | Antisense primer for *hdeB* (YPK_1140) | |
| IV961 | | GGTTATGAGATATTGTCACGCC | | Sense primer *cnfY* (YPK_2615) | |
| IV962 | | CGTTCCTCTCATTAGAATTACCG | | Antisense primer for *cnfY* (YPK_2615) | |
| IV966 | | GCCTTTCCATGACCTGCCCC | | Antisense primer for *ail* (YPK_1268) | |
| IV967 | | CTGATTGACGTTAATCTCGGCG | | Sense primer for *uspA* (YPK_0120) | |
| IV968 | | CTCCAGAAATCCTGATGATGACC | | Antisense primer for *uspA* (YPK_0120) | |
| V4 | | TGATTGGCGATGAGGTTACGG | | Sense primer for *csrA* (YPK_3372) | |
| V5 | | TTCTGCTTGGATGCGCTGGT | | Antisense primer for *csrA* (YPK_3372) | |
| V56 | | TGCGGCTGGCACTAAAGACA | | Sense primer for *yadA* (pYV0013) | |
| V57 | | TTTGGCCGCATCCAAAACAT | | Antisense primer for *yadA* (pYV0013) | |
| V588 | | CGTGAAAGGCTCCGTTGCGG | | Sense primer for *crp* (YPK_0248) | |
| V589 | | GTAAGAAATTTCAGCCACTTCAC | | Antisense primer for *crp* (YPK_0248) | |
| VI863 | | GGTGGCTCAAACGCTCAAGA | | Sense primer for *yscF* (pYV0082) | |
| VI864 | | GTAAGTCAGCAAGTAGCGCCG | | Antisense primer for *yscF* (pYV0082) | |
| VI865 | | CCGGGGGTTCGAGGAATCTG | | Sense primer for *ail* (YPK_1268) | |
| VI866 | | GCCCGCATTGGTAATCCAGG | | Sense primer for *yopJ* (pYV0098) | |
| VI867 | | CCCCCATGTTAATTATGAAGCGGG | | Antisense primer for *yopJ* (pYV0098) | |
| VI868 | | CCTGACTCAGGCTGCACGTA | | Sense primer for *fliC* (YPK_2381) | |
| VI869 | | CGCAGGCTGATTTCATCCTGA | | Antisense primer for *fliC* (YPK_2381) | |
| VI870 | | GCGGCATCGAAACCAATCCG | | Sense primer for *frdA* (YPK_3813) | |
| VI871 | | GTAAAGCGGCTTGTTCACCGG | | Antisense primer for *frdA* (YPK_3813) | |
| VI872 | | CGCTGAGTTTGACCCGGAAA | | Sense primer for *rfaH* (YPK_3937) | |
| VI873 | | CAGCAATGACGGTTGCTGGG | | Antisense primer for *rfaH* (YPK_3937) | |
| VI874 | | CGGCCCAAGTTTGGAACCAT | | Sense primer for *sodB* (YPK_1863) | |
| VI875 | | GGCAGCATCAGTAAATTGCGCT | | Antisense primer for *sodB* (YPK_1863) | |
| VI893 | | CTGCCAGGCAAAAATGGCTTG | | Sense primer for *arcA* (YPK_3606) | |
| VI894 | | CAGGTTACGGGCACGGATG | | Antisense primer for *arcA* (YPK_3606) | |
| VI895 | | GGTTTCCATCTGGCGGGTGA | | Sense primer for *fnr* (YPK_1944) | |
| VI896 | | GCGCAGATTGGGCATTTTACCG | | Antisense primer for *fnr* (YPK_1944 | |
| VI897 | | CGACCGCGGTCGGTATGTTC | | Sense primer for *napA* (YPK_1387) | |
| VI898 | | ACCGAGGACGCCATACAATGG | | Antisense primer for *napA* (YPK_1387) | |
| VI899 | | GCTTCGGTTTCATCACTCCAGC | | Sense primer for *cspC* (YPK_2474) | |
| VI900 | | GGCCTTCAGCCAAGGTTTTGAAG | | Antisense primer for *cspC* (YPK_2474) | |
| VI901 | | CCGTCCTGGTACCGATGATGG | | Sense primer for *if-3* (YPK_1821) | |
| VI902 | | CATTTCACGCCCACGGAACC | | Antisense primer for *if-3* (YPK_1821) | |
| IV122 | | ATCTGGCCGGGCGCACTG | | Sense primer for *yopE* (pYV0025) | |
| IV123 | | TTGGCAGCGTCTCAGCAGC | | Antisense primer for *yopE* (pYV0025) | |
| IV779 | | CGCCAAAGTGCTGTTGGAGG | | Sense primer for *yopE* (pYV0025) | |
| IV780 | | CGCTTCTGGGCCATAAGGAG | | Antisense primer for *yopE* (pYV0025) | |
| IV955 | | GGTAGCGGAGATGGTCAGCG | | Sense primer for *yopE* (pYV0025) | |
| IV956 | | CACCAATGTTACTCATTTGCTGC | | Antisense primer for *yopE* (pYV0025) | |
| VII771 | | GGAGCTTGGAGTAGGAAACC | | Sense primer for *yopE* (pYV0025) | |
| VII772 | | CTCCAGCTATACCAGGTCGA | | Antisense primer for *yopE*  (pYV0025) | |
|  |  | |  | |  |

Restriction sites are underlined and given in bold.
